# ALDH2 in autophagy and cell death: molecular mechanisms and implications for diseases

**DOI:** 10.1186/s40779-025-00646-8

**Published:** 2025-09-18

**Authors:** Yu Duan, Ze-Chen Shan, Jiao-Jiao Pang, Yu-Guo Chen

**Affiliations:** 1 Department of Emergency Medicine, Qilu Hospital of Shandong University, Jinan, 250000 China; 2 Chest Pain Center, Shandong Provincial Clinical Research Center for Emergency and Critical Care Medicine, Institute of Emergency and Critical Care Medicine of Shandong University, Qilu Hospital of Shandong University, Jinan, 250000 China; 3 Key Laboratory of Emergency and Critical Care Medicine of Shandong Province, Key Laboratory of Cardiopulmonary-Cerebral Resuscitation Research of Shandong Province, Shandong Provincial Engineering Laboratory for Emergency and Critical Care Medicine, Qilu Hospital of Shandong University, Jinan, 250000 China; 4 The Key Laboratory of Cardiovascular Remodeling and Function Research, Chinese Ministry of Education, Chinese Ministry of Health and Chinese Academy of Medical Sciences, the State and Shandong Province Joint Key Laboratory of Translational Cardiovascular Medicine, Qilu Hospital of Shandong University, Jinan, 250000 China

**Keywords:** Aldehyde dehydrogenase (ALDH) 2, Autophagy, Cell death, Human diseases

## Abstract

Aldehyde dehydrogenase (ALDH) 2, a mitochondrial enzyme, is the main acetaldehyde dehydrogenase involved in the scavenging of alcohol-derived acetaldehyde and endogenous aldehydes. The *ALDH2*^rs671^ mutation affects 560 million East Asians and is closely related to an increased risk of various human diseases. In addition to its well-known function in detoxifying alcohol-derived acetaldehyde and endogenous aldehydes, ALDH2 is implicated in human health through its regulation of autophagic machinery and multiple cell death pathways (e.g., apoptosis, necroptosis, pyroptosis, ferroptosis, and NETosis). This review summarizes the current knowledge of ALDH2 and the regulatory mechanism through which ALDH2 regulates autophagy and cell death. In addition, we outline the potential role of ALDH2 in the regulation of autophagy and cell death during the occurrence and progression of human diseases, aiming to provide a novel theoretical framework for human disease treatment.

## Background

Cell death manifests in diverse forms, and the original objective of its activation is to cope with physiological or pathological processes, including normal tissue development, infection, and immune surveillance [1]. However, in some cases, cell death is overactivated, which can not only lead to the release of multiple inflammatory mediators but also further amplify the inflammatory response and aggravate organ damage through damage-associated molecular patterns, resulting in the occurrence or deterioration of various human diseases, such as cancer, sepsis, and autoimmune diseases [1]. In addition, during sepsis or human immunodeficiency virus infection, immune cell death is closely associated with the immunoparalysis of patients, which is a key factor contributing to the high mortality rate in these diseases [2, 3]. Like cell death, autophagy, a homeostatic catabolic process, can be initiated in a variety of ways in response to different physiological or pathological stimuli [4, 5]. However, uncontrolled or excessive autophagy activation also leads to cell death [1]. Given the importance of autophagy and cell death in regulating cellular homeostasis and survival, a better understanding of the key regulators that modulate the physiological and mechanistic aspects of these processes may yield improved treatments for addressing unmet medical needs.

Aldehyde dehydrogenase (ALDH) is a protein superfamily that contains a total of 19 ALDH isoenzymes whose expression, structures, and functions differ across tissues [6, 7]. The Main function of the ALDH isoform is to detoxify ethanol-derived acetaldehyde and endogenous Lipid aldehydes such as 4-hydroxy-2-nonenal (4-HNE) or malondialdehyde [6]. Notably, ALDH2 is more efficient at clearing acetaldehyde than other members of the family due to its high enzyme affinity. Therefore, in humans, ALDH2 is the major ALDH enzyme involved in cellular acetaldehyde metabolism [7]. Accumulating evidence has demonstrated that *ALDH*2*2, a variant allele marked by the *ALDH2* polymorphism rs671, is highly prevalent in East Asian populations and has significantly decreased enzyme activity [8]. Reduced ALDH2 activity is critically involved in a wide range of human diseases, ranging from inflammatory disease to tumorigenesis [7, 8]. However, the accumulation of reactive aldehydes cannot completely explain the occurrence and progression of these diseases, indicating that additional regulatory mechanisms of ALDH2 in human diseases remain to be elucidated.

Beyond the canonical role of ALDH2 in mediating aldehyde clearance, emerging evidence highlights the function of ALDH2 in regulating autophagy and cell death. Thus, in this review, we outline the regulatory mechanism of ALDH2 in autophagy and cell death and elucidate its role in human diseases, aiming to provide a novel framework for understanding the biological role of ALDH2 in health and disease.

## The ALDH superfamily

**Fig. 1:**
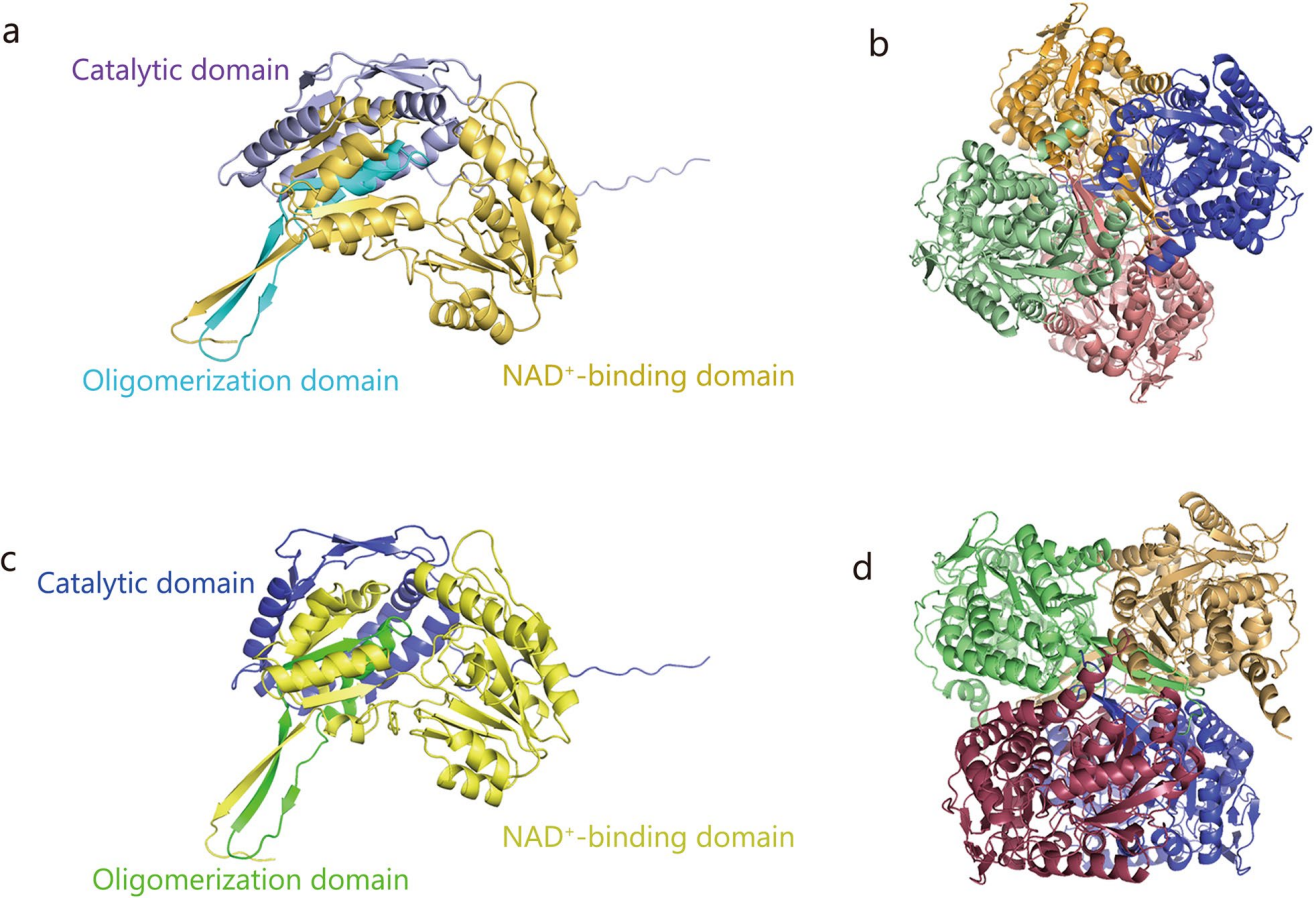
ALDH architecture. **a** Three conserved domains in an ALDH monomer (mouse): catalytic (purple), NAD(P) binding (yellow), and oligomerization (blue). **b** Homotetrameric structure of ALDH2 (mouse), individual subunits are rendered in different colors (yellow, purple, pink, green). **c** Three conserved domains in an ALDH monomer (human): catalytic (purple), NAD(P) binding (yellow), and oligomerization (green). **d** Homotetrameric structure of ALDH2 (human), individual subunits are rendered in different colors (yellow, purple, red, green). ALDH aldehyde dehydrogenase, NAD^+^ nicotinamide adenine dinucleotide

The ALDH superfamily comprises 19 nicotinamide adenine dinucleotide (NAD^+^)-dependent oxidoreductases that share a conserved catalytic zinc (II) center coordinated by 2 cysteine residues and 1 histidine [9, 10]. The typical subunit size of the ALDH family of enzymes is approximately 50 kD and contains 450–500 amino acid residues. Structurally, each subunit comprises 3 conserved domains, including the catalytic domain, the coenzyme- or NAD^+^-binding domain, and the oligomerization domain (Fig. 1). The members of this superfamily include ALDH1 (1A1, 1A2, 1A3, 1B1, 1L1, and 1L2), ALDH2, ALDH3 (3A1, 3A2, 3B1, and 3B2), ALDH4A1, ALDH5A1, ALDH6A1, ALDH7A1, ALDH8A1, ALDH9A1, ALDH16A1, and ALDH18A1 [9, 10]. In addition to their primary catalytic role, ALDH isoforms exhibit diverse biological functions, including detoxification, biosynthesis, antioxidant function, and regulation of cellular processes. Additionally, they exhibit significant differences in terms of their subcellular localization, tissue distribution, and physiological functions, as summarized in Table 1 [6, 11–32].

**Table 1:** ALDH isoforms: distribution, substrates, and functions

Isoform	Subcellular distribution	Major substrate	Physiological role	References
1A1	Cytosol	Retinal	Retinoic acid metabolism	[11]
1A2	Cytosol	Retinal	Retinoic acid metabolism	[12]
1A3	Cytosol	Retinal	Retinoic acid metabolism	[13]
1B1	Cytosol	Acetaldehyde	Corneal UV protection	[14]
1L1	Mitochondria	10-fTHF	Folate metabolism	[15]
1L2	Cytosol	10-fTHF	Folate metabolism	[16]
2	Mitochondria, ER, Cytosol	Acetaldehyde, cholesterol	Alcohol and cholesterol metabolism	[6]
3A1	Cytosol, nucleus	Aliphatic, aromatic aldehydes	Regulation of cell proliferation	[17, 18]
3A2	Microsomes, peroxisomes	Fatty aldehydes	Fatty aldehyde oxidation	[19]
3B1	Mitochondria	Octanal	Fatty aldehyde oxidation	[20, 21]
3B2	Mitochondria	LPO-derived aldehydes	Lipid peroxide scavenging	[22]
4A1	Mitochondria	γ-glutamate semialdehyde	Glutamate metabolism	[23]
5A1	Mitochondria	Succinate semialdehyde	GABA metabolism	[24, 25]
6A1	Mitochondria	Malonate semialdehyde	Valine and pyrimidine metabolism	[26, 27]
7A1	Mitochondria, cytosol	Aminoadipic semialdehyde	Lysine metabolism	[28]
8A1	Cytosol	Retinal	Kynurenine pathway	[29]
9A1	Cytosol	γ-amino butyraldehyde	GABA and DOPAL metabolism	[30]
16A1	Unknown	Unknown	Uric acid metabolism in the kidneys	[31]
18A1	Mitochondria	γ-glutamate semialdehyde	Proline metabolism	[32]

*ALDH* aldehyde dehydrogenase, *10-fTHF* 10-formyltetrahydrofolate, *DOPAL* 3,4-dihydroxyphenylacetaldehyde, *ER* endoplasmic reticulum, *GABA* gamma-aminobutyric acid, *LPO* lipid peroxidation, *UV* ultraviolet

## ALDH2 structure, location, and function

ALDH2 is a tetrameric protein consisting of 517 amino acids and 4 identical subunits [6] **(**Fig. 1). As noted earlier, each subunit contains catalytic, NAD⁺-binding, and oligomerization domains [6, 33, 34]. The entrance of ALDH2 into the mitochondrial Matrix relies on its N-terminal 17-amino acid targeting sequence, which is eliminated following the formation of a functionally active homotetramer [35]. ALDH2 activity is dependent on its cofactor NAD⁺, and the interaction between the two directly affects the efficiency of aldehyde metabolism [36]. Specifically, ALDH2 catalyzes the oxidation of acetaldehyde to acetate using NAD⁺ as an electron acceptor, generating NADH during this process. Furthermore, NAD⁺ can bind to the allosteric site of ALDH2 to stabilize its tetrameric structure, thereby enhancing its enzymatic activity [36]. It has been confirmed that the mutant *ALDH2* allele (ALDH2*2) is a predominant genetic polymorphism in Human populations. Substitution of glutamic acid by lysine at position 504 (Glu504Lys) caused by a single-point mutation (rs671) results in the ALDH2*2 variant. The mutation of rs671 is localized mainly within the ALDH2 oligomerization domain, which causes a conformational change in ALDH2 and disrupts the active site and its coenzyme-binding domain, contributing to dramatically reduced ALDH2 activity [37, 38]. Mechanistically, ALDH2 amino acid residues 463–478 bind with residues 269–272 through 2 hydrogen bonds. The interaction of the active site and residues 269–272 relies on a hydrogen bond between the side chain of Glu399 and the peptide nitrogen at position 271. Glu399 forms a hydrogen bond between its carboxylate group and the 2’ and 3’ hydroxyl oxygens of nicotinamide ribose, which in turn stabilizes the nicotinamide portion of NAD^+^. *ALDH2*^rs671^, however, can disrupt the above interactions and affect tetramer formation, resulting in the inhibition of the nucleophilic attack on aldehydes and impairment of the transfer of hydrogen ions to NAD^+^. Therefore, compared with normal people (ALDH2*1/1), heterozygous individuals (ALDH2*1/2) have significantly decreased enzyme activity (< 50%), while homozygous variant (ALDH2*2/2) carriers exhibit much lower activity (< 5%) [37, 38].

ALDH2 can be found in a variety of tissues and is abundantly expressed in the liver, affecting multiple regulatory pathways [39]. ALDH2 is located mainly in the mitochondria but is expressed in the cytoplasm, endoplasmic reticulum (ER), and nucleus [33, 40–42]. Notably, different ALDH2 locations have distinct functions. Mitochondrial ALDH2 predominantly participates in cell metabolism pathways, including ethanol metabolism and the clearance of endogenous reactive aldehydes generated from lipid peroxidation [33]. ALDH2 in the cytoplasm and ER plays a nonenzymatic role in cholesterol metabolism. Mechanistically, cytoplasmic ALDH2 can bind to poly(ADP-ribose) polymerase 1, leading to the inhibition of poly(ADP-ribose) polymerase 1 translocation from the cytoplasm to the nucleus, which in turn suppresses the poly(ADP-ribosyl)ation of liver X receptor-α and enhances high-density lipoprotein biogenesis. However, the molecular mechanism underlying ALDH2 relocalization to the cytoplasm during this process is not fully understood [40]. 3-Hydroxy-3-methylglutaryl-coenzyme A reductase (HMGCR), a rate-limiting enzyme in cholesterol synthesis, interacts with ALDH2 in the ER, which in turn triggers ubiquitination-mediated degradation of HMGCR to limit cholesterol synthesis. In this process, the translocation of ALDH2 from mitochondria to the ER is essential for its interaction with HMGCR. Treatment with 1-methyl-4-phenylpyridinium, which blocks the above translocation, significantly attenuates the ALDH2-HMGCR interaction [41]. In addition, nuclear ALDH2 has been shown to regulate lysosomal homeostasis through transcriptional regulation of ATPase H^+^ Transporting V0 Subunit E2 (*ATP6V0E2)*. Mechanistically, the *ALDH2*^rs671^ mutation disrupts the low-density lipoprotein receptor-AMP-activated protein kinase (AMPK) interaction, leading to AMPK activation. Activated AMPK then phosphorylates mitochondrial ALDH2, promoting its translocation from the mitochondria to the nucleus [42].

In summary, these findings show that the localization of ALDH2 is closely related to its function and regulatory signals. Further exploration of the cellular localization of ALDH2 under different physiological or pathological conditions might provide new therapeutic strategies for human diseases.

## ALDH2 and autophagy

Autophagy can be divided into 3 main types: macroautophagy, microautophagy, and chaperone-mediated autophagy [5]. Macroautophagy (hereafter called autophagy) is the most studied form of autophagy and can modulate cell homeostasis through multiple pathways, such as the supplementation of extra energy, elimination of aberrant protein deposits, and regulation of cell death [4]. Therefore, autophagy plays multiple roles in physiological and pathological processes, including cell growth and death, immune responses, and inflammation [4, 5]. Owing to the importance of autophagy, it is considered a novel avenue for preventing or treating many diseases. Here, we summarize the basic molecular mechanism of autophagy and discuss in depth the relationship between ALDH2 and autophagy.

### Molecular mechanism of autophagy

**Fig. 2:**
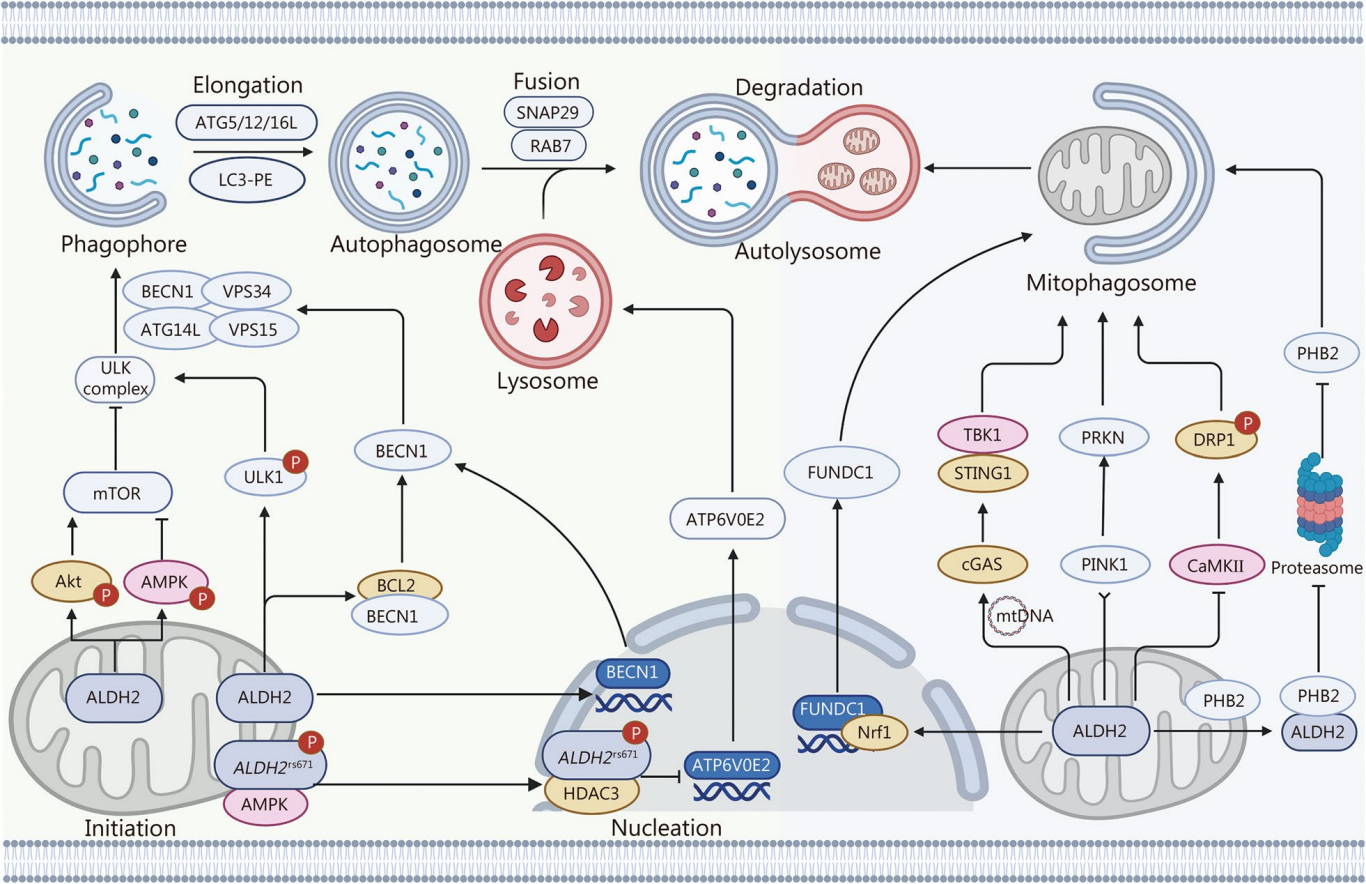
ALDH2 in autophagy. ALDH2 initiates autophagy by phosphorylating AMPK and inhibiting mTOR activation. ALDH2 promotes the ULK1 phosphorylation to initiate autophagy. ALDH2 facilitates Akt phosphorylation and promotes mTOR activation to suppress autophagy. ALDH2 upregulates the expression of BECN1 and releases BECN1 from the complex to promote autophagy. AMPK phosphorylates *ALDH2*^rs671^ and promotes its nuclear translocation. In turn, *ALDH2*^rs671^ binds to HDAC3 to suppress *ATP6V0E2* transcription and inhibit autophagic fusion. ALDH2 promotes FUNDC1-mediated mitophagy via Nrf1. ALDH2 activation allows mtDNA leakage into the cytosol, activating the cGAS-STING-TBK1 pathway to promote mitophagy. ALDH2 initiates mitophagy or inhibits excessive mitophagy activation through the PINK-PRKN pathway. ALDH2 inhibits mitophagy by inhibiting the CaMKII-DRP1 axis. ALDH2 interacts with PHB2 to inhibit its proteasomal degradation, thereby promoting mitophagy. Akt threonine-serine protein kinase B, ALDH2 aldehyde dehydrogenase 2, AMPK AMP-activated protein kinase, ATG autophagy-related, ATP6V0E2 ATPase H^+^ Transporting V0 Subunit E2, BCL2 B-cell lymphoma 2, BECN1 Beclin1, CaMKII Calmodulin-dependent kinase II, cGAS Cyclic guanosine monophosphate-adenosine monophosphate synthase, DRP1 dynamin-related protein 1, FUNDC1 FUN14 domain containing 1, HDAC3 histone deacetylase 3, LC3-PE microtubule-associated protein Light chain 3-Phosphatidylethanolamine, mTOR mechanistic target of rapamycin, Nrf1 nuclear respiratory factor 1, PHB2 Prohibitin 2, PINK PTEN-induced putative kinase 1, PRKN parkin RBR E3 ubiquitin protein ligase, RAB7 ras-related protein Rab-7a, SNAP29 synaptosomal-associated protein of 29 kD, STING stimulator of interferon genes, TBK1 TanK-binding kinase 1, ULK Unc-51-like kinase, VPS vacuolar protein sorting

The classical autophagic pathway can Generally be divided into 5 steps, namely, initiation, nucleation, elongation, fusion, and degradation (Fig. 2). The initiation step is induced by the inhibition of mechanistic target of rapamycin (mTOR), which contributes to the activation of the unc-51-like kinase (ULK) complex, including ULK1, focal adhesion kinase (FAK) family kinase-interacting protein (200 kD), autophagy-related (ATG)13, and ATG101. The beclin1 (BECN1)-vacuolar protein sorting 34 (VPS34) complex, whose major components are BECN1, VPS34, VPS15, and ATG14L, is subsequently involved in the nucleation process and phagophore formation. Furthermore, the elongation step and autophagosome formation are completed through 2 ubiquitin-like conjugation systems, ATG5-ATG12-ATG16L and microtubule-associated protein Light chain 3-phosphatidylethanolamine (LC3-PE). Finally, the mature autophagosome fuses with the lysosome to form the autolysosome, leading to degradation of the contents. Notably, functional lysosomes, certain cytoskeleton motor proteins, tethering factors, soluble N-ethylmaleimide sensitive factor attachment protein receptor proteins, and phospholipids, such as the ras-related protein Rab-7a and synaptosomal-associated protein (29 kD), play a fundamental role in the fusion and degradation phases [43].

### Regulation of autophagy by ALDH2

Numerous studies have shown that ALDH2 regulates autophagy through multiple pathways (Fig. 2). First, ALDH2 can initiate autophagy by promoting the phosphorylation of AMPK and inhibiting mTOR activation [44, 45]. ALDH2 is activated to increase ULK1 phosphorylation at serine (Ser)^555^ and Ser^317^ and promote the initiation of autophagy [46]. Intriguingly, studies have shown that ALDH2 also facilitates the phosphorylation of threonine-serine protein kinase B (Akt) and promotes mTOR activation, thus inhibiting the initiation of autophagy [44, 47]. Second, researchers have reported that BECN1 is a target of ALDH2 in regulating the autophagic nucleation process [48, 49]. Mechanistically, ALDH2 can not only promote autophagic flux by upregulating the expression of BECN1 but also release BECN1 to participate in the autophagic process by interfering with the interaction between BECN1 and B-cell lymphoma 2 (BCL2) [48, 49]. Third, the activation of AMPK caused by the *ALDH2*^rs671^ mutation has been confirmed to promote the translocation of ALDH2 from the mitochondria to the nucleus through the phosphorylation of ALDH2 at threonine (Thr)356 [42]. ALDH2 in the nucleus subsequently interacts with histone deacetylase 3 (HDAC3), which in turn inhibits the transcription of the lysosomal protein ATP6V0E2, thus impairing lysosomal function and inhibiting the autophagic fusion process [42]. In summary, these data indicate that ALDH2 regulates autophagy through initiation, nucleation, and fusion, and whether ALDH2 can regulate autophagy through other pathways requires further investigation.

In addition to nonselective autophagy, ALDH2 regulates selective autophagy (Fig. 2). Mitophagy, the selective degradation of mitochondria via the autophagic machinery, is modulated by ALDH2 [4]. It has been reported that functional ALDH2 activation allows the release of a small amount of mitochondrial DNA (mtDNA), which in turn activates the cyclic guanosine monophosphate-adenosine monophosphate synthase (cGAS)-stimulator of interferon genes (STING)-TANK-binding kinase 1 (TBK1) pathway, initiating mitophagy to clear damaged mitochondria [50]. In a recent report, ALDH2 was shown to initiate mitophagy by regulating the activation of nuclear respiratory factor 1, which directly binds to the promoter of the mitophagy receptor FUN14 domain-containing 1 (FUNDC1) and upregulates FUNDC1 expression [51]. Li et al. [52] revealed that ALDH2 interacts with the mitophagy receptor prohibitin 2 (PHB2) to impair the proteasomal degradation of PHB2, thereby enhancing mitophagy. Researchers have reported that the inhibition of ALDH2 activation leads to the impairment of PTEN-induced putative kinase 1 (PINK1)-Parkin RBR E3 ubiquitin protein ligase (PRKN)-dependent mitophagy, which can be reversed by Alda-1, an activator of ALDH2 [53, 54]. Notably, some studies have shown that ALDH2 is also capable of suppressing excessive mitophagy mediated by the PINK1-PRKN pathway to maintain necessary energy metabolism [55–57]. ALDH2 deficiency results in the activation of calmodulin-dependent kinase II (CaMKII) and the promotion of dynamin-related protein 1 (DRP1) phosphorylation, which in turn induces the overactivation of mitophagy [58]. Thus, these findings suggest that ALDH2 might balance the number of mitochondria to maintain cell homeostasis via autophagic machinery, but the exact mechanism remains to be elucidated.

Other types of selective autophagy are rarely reported to be regulated by ALDH2. Although ALDH2 dysfunction also causes aberrant function in organelles, such as excess ER stress and lipid metabolism dysregulation, the underlying mechanism has not yet been elucidated [59, 60]. Similarly, the autophagic machinery plays a vital role in regulating the abovementioned dysfunctional processes [43]. Thus, clarifying the potential roles of ALDH2 and autophagy in these processes may lead to the identification of new targets for therapeutic interventions. Notably, autophagy-dependent cell death (ADCD) is a type of regulated cell death (RCD) that mechanistically relies on the autophagic machinery. The term ADCD should not refer to settings in which the autophagic apparatus is merely activated alongside (rather than precipitating) RCD or in which autophagy promotes other RCD modalities, such as ferroptosis through ferritinophagy (autophagic degradation of ferritin) [1, 4]. Despite the well-documented connections between ALDH2 and autophagic regulation, direct evidence for ALDH2-mediated regulation of ADCD is currently lacking, representing a critical knowledge gap in the field.

## ALDH2 in cell death

**Fig. 3:**
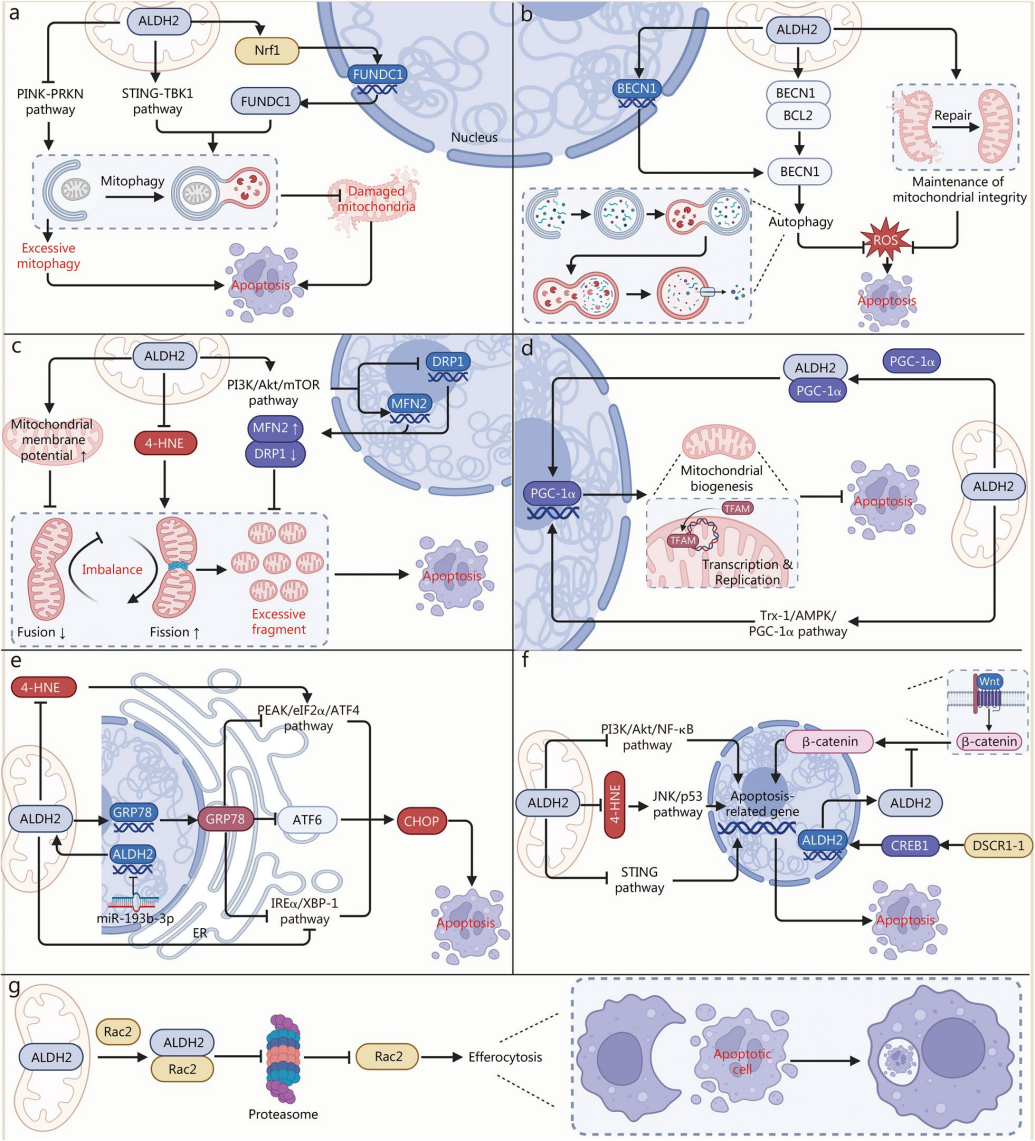
ALDH2 in apoptosis. **a** ALDH2 inhibits excessive mitophagy to suppress apoptosis through the PINK-PRKN pathway. ALDH2 activates the STING-TBK1 pathway or promotes the Nrf-FUNDC1 axis to initiate mitophagy, thereby inhibiting apoptosis. **b** ALDH2 maintains mitochondrial integrity and reduces ROS to prevent apoptosis. ALDH2 enhances BECN1 expression and release to promote autophagy, thereby reducing ROS and apoptosis. **c** ALDH2 elevates mitochondrial membrane potential to promote mitofusion (MFN) and suppress apoptosis. ALDH2 inhibits 4-HNE accumulation and excessive mitofission, alleviating apoptosis. ALDH2 suppresses apoptosis via PI3K/Akt/mTOR-mediated MFN2 upregulation and DRP1 downregulation. **d** ALDH2-PGC-1α axis promotes mitobiogenesis and inhibits apoptosis. ALDH2 activates Trx-1/AMPK/PGC-1α pathway to enhance mitobiogenesis and inhibit apoptosis. **e** ALDH2 blocks 4-HNE-induced PERK/eIF2α/ATF4 activation to alleviate ER stress and apoptosis. ALDH2 inhibits the IREα/XBP-1 pathway or upregulates GRP78 expression to inhibit ER stress-related apoptosis. MiR-193b-3p promotes apoptosis via ALDH2 inhibition and ER stress activation **f** ALDH2 suppresses apoptosis by inhibiting PI3K/Akt/NF-κB and STING pathways. ALDH2 blocks 4-HNE-induced JNK/p53 activation to suppress apoptosis. DSCR1-1 enhances ALDH2 transcription via CREB1 nuclear translocation, leading to inhibition of Wnt/β-catenin signaling and apoptosis suppression. **g** ALDH2-Rac2 axis enhances efferocytosis by inhibiting Rac2 degradation. 4-HNE 4-hydroxy-2-nonenal, Akt threonine-serine protein kinase B, ALDH2 aldehyde dehydrogenase 2, AMPK AMP-activated protein kinase, ATF4/6 Activating transcription factor 4/6, ATP6V0E2 ATPase H^+^ Transporting V0 Subunit E2, BCL2 B-cell lymphoma 2, BECN1 Beclin1, cGAS Cyclic guanosine monophosphate-adenosine monophosphate synthase, CHOP C/EBP homologous protein, CREB1 cAMP-responsive element protein 1, DRP1 dynamin-related protein 1, DSCR1-1 down syndrome candidate region 1–1, eIF2α eukaryotic initiation factor 2 alpha, ER endoplasmic reticulum, FUNDC1 FUN14 domain containing 1, GRP78 glucose-regulating protein 78, IREα inositol requiring kinase 1α, JNK c-Jun N-terminal kinase, LC3-PE microtubule-associated protein Light chain 3-phosphatidylethanolamine, mTOR mechanistic target of rapamycin, NF-κB nuclear factor kappa B, Nrf1 nuclear respiratory factor 1, PERK Pancreatic ER kinase (PKR)-like ER kinase, PGC-1α peroxisome proliferator-activated receptor gamma coactivator 1 alpha, PI3K phosphatidylinositol 3-kinase, PINK PTEN-induced putative kinase 1, PRKN parkin RBR E3 ubiquitin protein ligase, Rac1 ras-related C3 botulinum toxin substrate, ROS reactive oxygen species, STING stimulator of interferon genes, TBK1 TanK-binding kinase 1, TFAM mitochondrial transcription factor A, Trx-1 thioredoxin-1, ULK Unc-51-like kinase, Wnt wingless and int-1, XBP-1 X-box binding protein-1

**Fig. 4:**
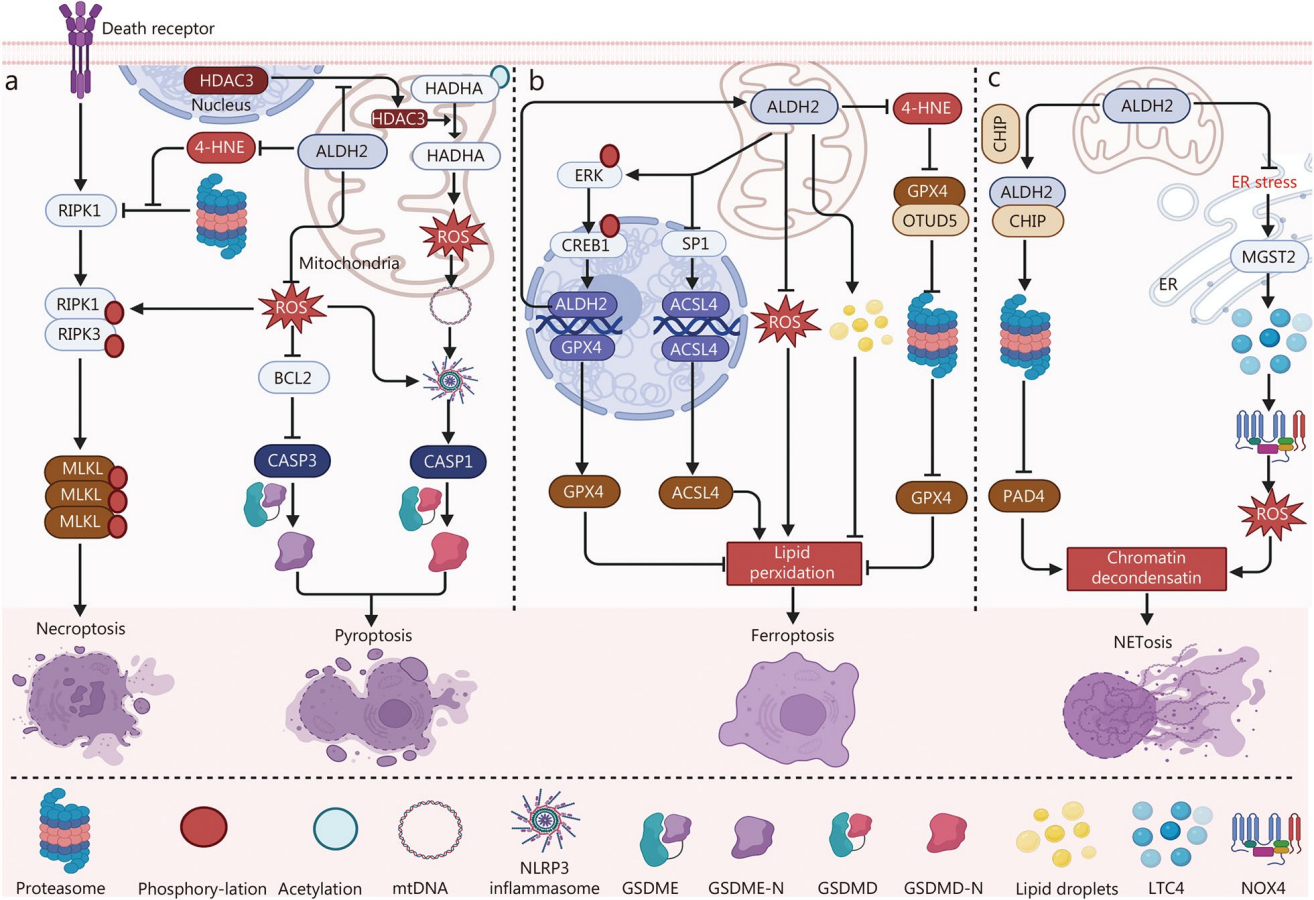
ALDH2 in necroptosis, pyroptosis, ferroptosis, and NETosis. **a** ALDH2 reduces ROS to suppress necroptosis. ALDH2 blocks 4-HNE-induced necroptosis by promoting RIPK1 degradation. ALDH2 blocks HDAC3 mitochondrial translocation and subsequent HADHA deacetylation, suppressing the ROS-mtDNA-NLRP3 axis to attenuate pyroptosis. ALDH2 reduces ROS to suppress both BCL2-CASP3-GSDME and NLRP3-CASP1-GSDMD-mediated pyroptosis. **b** ALDH2 activates ERK/CREB1 signaling to upregulate GPX4 and ALDH2 expression, thereby inhibiting ferroptosis. ALDH2 suppresses ACSL4 expression by inhibiting SP1 activation to attenuate ferroptosis. ALDH2 mitigates ferroptosis via ROS reduction and lipid droplet biogenesis. ALDH2 counteracts 4-HNE-induced GPX4 degradation by preserving the GPX4-OTUD5 interaction, thereby inhibiting ferroptosis. **c** ALDH2-CHIP interaction inhibits NETosis by promoting PAD4 degradation. ALDH2 suppresses NETosis via ER stress-MGST2-LTC4-NOX2 cascade. 4-HNE 4-hydroxy-2-nonenal, ALDH2 aldehyde dehydrogenase 2, ACSL4 Acyl-CoA synthetase Long-chain family member 4, BCL2 B-cell lymphoma 2, CASP1/3 caspase 1/3, CHIP C-terminus of HSC70-interacting protein, CREB1 cAMP-responsive element protein 1, ER endoplasmic reticulum, ERK extracellular signal-regulated kinase, GSDMD gasdermin-D, GSDME gasdermin-E, HADHA hydroxyl-CoA dehydrogenase alpha subunit, HDAC3 histone deacetylase 3, GPX4 glutathione peroxidase 4, LTC4 leukotriene C4, MGST2 microsomal glutathione S-transferase 2, MLKL mixed lineage kinase domain-like protein, mtDNA mitochondrial DNA, NLRP3 nucleotide-binding domain (NBD), leucine-rich repeat (LRR), and pyrin, NOX4 NADPH oxidases 4, OTUD5 ovarian tumor (OTU) deubiquitinase 5, PAD4 peptidylarginine deiminase 4, RIPK1/3 receptor-interacting protein kinase 1/3, ROS reactive oxygen species, SP1 specificity protein 1

Currently, according to the novel definition, cell death can be divided into RCD and accidental cell death. Accidental cell death is a form of uncontrolled process that is characterized by overwhelming cell injury and an unregulated mechanism. Conversely, RCD, also known as programmed cell death, is an adjustable process and involves precise regulatory mechanisms. Among them, programmed cell death refers to RCD that occurs under physiological conditions without exogenous environmental perturbation. Therefore, RCD is critically involved in a variety of physiological or pathological processes, such as immune responses and cell development [1]. Recently, emerging evidence has shown that ALDH2 plays a vital role in regulating cell death. In this section, we outline the regulatory mechanism through which ALDH2 modulates multiple forms of RCD, including apoptosis, necroptosis, pyroptosis, ferroptosis, and NETosis (Figs. 3, 4).

### Apoptosis

Apoptosis refers to an immune-silent form of programmed cell death due to the limited release of cellular content, which can be Quickly eliminated. It has been confirmed that the initiation of apoptosis occurs through 2 distinct pathways, the death receptor (extrinsic) pathway and the mitochondrial (intrinsic) pathway. Caspase (CASP) family proteins are critically involved in this process because CASP3/7 are the executors of apoptosis, and their activation relies on cleavage mediated by other CASP proteins. Under lethal stimuli, elevated mitochondrial outer membrane permeability allows the release of mitochondrial proteins, initiating the mitochondrial apoptosis pathway. Mitochondrial proteins in the cytosol subsequently interact with the adaptor and form an apoptosome, which in turn activates CASP3/7 to trigger apoptosis. Activation of the death receptor pathway requires binding between the cell surface death receptor and its ligand, which can lead to CASP8 activation. Finally, CASP8 cleaves and activates CASP3/7 to induce apoptosis [1].

#### Mitophagy

Damage to mitochondria promotes the leakage of contents, which is detrimental to the cell and even activates the mitochondrial apoptosis pathway [1]. Therefore, the clearance of damaged mitochondria is vital for maintaining cell viability. ALDH2-mediated mitophagy plays a broad role in regulating apoptosis sensitivity (Fig. 3a). For example, FUNDC1-mediated mitophagy is required for rescuing pressure overload-induced cardiomyocyte apoptosis through transcriptional upregulation of FUNDC1 in an ALDH2-nuclear respiratory factor 1 (Nrf1)-dependent manner [51]. The ALDH2-STING-TBK1-mitophagy axis is involved in modulating apoptosis. Mechanistically, ALDH2 can preserve mitochondrial integrity and allow the release of low doses of mtDNA into the cytosol, sustaining the activation of the STING pathway. TBK1 phosphorylation, activated by the STING pathway, subsequently facilitates mitophagy to remove defective mitochondria and maintain cell viability [50]. In contrast, excessive mitophagy has been confirmed to induce apoptosis in lipopolysaccharide (LPS)-induced myocardial injury, and Alda-1 treatment or ALDH2 overexpression can alleviate cell death by suppressing PINK-PRKN-dependent mitophagy [55]. In general, ALDH2 regulates apoptosis through 2 main pathways: the activation of mitophagy to clear damaged mitochondria and the inhibition of excessive mitophagy. Notably, the mechanism by which ALDH2 balances mitophagy activation to regulate apoptosis remains unknown, and further studies are warranted.

#### Reactive oxygen species (ROS)

Mitochondria are the major organelles that produce ROS [61]. ROS are intimately associated with apoptosis because they cannot only induce apoptosis but also lead to mitochondrial damage to aggravate apoptotic pathway activation [1]. ALDH2 activation has been demonstrated to protect against neuronal apoptosis from lethal inflammation in an Alzheimer’s disease (AD) mouse model by preserving mitochondrial integrity and inhibiting ROS accumulation [62]. Calycosin, one of the major constituents of Radix Astragali, can eliminate intracellular ROS to alleviate oxidative stress-induced cardiomyocyte apoptosis by increasing ALDH2 activity and maintaining mitochondrial integrity [63]. In addition, Xu et al. [48] reported that ALDH2 limits cellular ROS levels by upregulating BECN1 expression and interrupting the interaction between BECN1 and BCL2 to release BECN1, contributing to the activation of autophagy and inhibition of apoptosis (Fig. 3b).

#### Mitochondrial dynamics

Mitochondrial dynamics are modulated by many GTPases located at mitochondrial membranes, such as mitofusion (MFN) 1/2, which activates fusion, and DRP1, which induces fission [61]. Excessive mitochondrial fragmentation caused by unbalanced mitofusion and mitofission is among the major contributors to apoptosis [64, 65]. In a recent report, the accumulation of 4-HNE and excessive mitofission were noted in cardiomyocytes from mice with streptozotocin-induced diabetes, along with evident apoptosis, which was caused by the defective activation of ALDH2 [66]. During ischemia/reperfusion (I/R) injury, Alda-1 can reduce cardiomyocyte apoptosis by increasing the mitochondrial membrane potential and promoting mitofusion [67]. Additionally, the activation of ALDH2 attenuates myocardial mitochondrial damage and apoptosis by Maintaining the balance of mitofusion and mitofission through the phosphatidylinositol 3-kinase (PI3K)/Akt/mTOR pathway, which upregulates MFN2 and downregulates DRP1 [68] (Fig. 3c).

#### Mitochondrial biogenesis

Mitochondrial biogenesis refers to the process by which new mitochondria are generated from existing mitochondria [61]. Peroxisome proliferator-activated receptor gamma coactivator 1 alpha (PGC-1α) plays a critical role in this process by directly modulating transcription factors and promoting biogenesis-related gene transcription [69]. In a recent article, ALDH2 was shown to interact with PGC-1α and accelerate its nuclear translocation, in turn promoting mitochondrial biogenesis and alleviating apoptosis [70]. Zhu et al. [71] revealed that ALDH2 alleviates myocardial apoptosis in myocardial I/R injury by activating the thioredoxin-1 (Trx-1)/AMPK/PGC-1α pathway and facilitating mitochondrial biogenesis (Fig. 3d).

#### ER stress

When ER function is perturbed, protein production exceeds its capacity, which in turn induces ER stress and the formation of unfolded or misfolded proteins [72, 73]. These proteins subsequently accumulate within the ER lumen and trigger the unfolded protein response (UPR) to restore ER homeostasis. Under normal conditions, glucose-regulating protein 78 (GRP78), a molecular chaperone, binds to UPR signaling receptors and suppresses their activation [72, 73]. In response to ER stress, unfolded proteins interact with GRP78 and promote its separation from receptors, activating relevant downstream signals, including activating transcription factor 6 (ATF6), pancreatic ER kinase (PKR)-like ER kinase (PERK)/eukaryotic initiation factor 2 alpha (eIF2α)/ATF4, and inositol-requiring enzyme 1 alpha (IREα)/X-box-binding protein 1 (XBP-1) [72, 73]. However, if the stress is persistent, the UPR exacerbates ER stress and triggers apoptosis through the activation of C/EBP homologous protein (CHOP)- and CASP9/12-dependent pathways [72, 73]. 4-HNE has been reported to mediate smooth muscle cell apoptosis by activating ER stress through the PERK/eIF2α/ATF4 signaling pathway, which can be mitigated by ALDH2 activation [74]. A reduction in miR‐193b‐3p was confirmed to alleviate oxidized low-density lipoprotein (ox-LDL)-induced human umbilical vein endothelial cell apoptosis through transcriptional upregulation of ALDH2 and suppression of ER stress [75]. Disturbed activation of ALDH2 triggers LPS-induced cardiomyocyte apoptosis by promoting ER stress through the IREα/XBP-1 pathway [59]. Ma et al. [76] reported that cardiac hypertrophic preconditioning plays a protective role against myocardial I/R injury by inhibiting ER stress-related apoptosis through an ALDH2-dependent mechanism. Additionally, during the aging process, ALDH2 enhances the UPR to improve the ability to modulate protein folding and rescue cells from apoptosis through the upregulation of GRP78 [77] (Fig. 3e).

#### Signaling pathways

Activation of the cGAS-STING pathway and apoptosis were observed in LPS-induced cardiac dysfunction, and these effects could be negated by treatment with Alda-1 [78]. Loss of ALDH2 activity triggers the accumulation of 4-HNE, resulting in cardiomyocyte apoptosis through the phosphorylation of c-Jun N-terminal kinase (JNK) and activation of p53 [79]. During the progression of osteoarthritis, down syndrome candidate region 1–1 (DSCR1-1) can inhibit chondrocyte apoptosis by regulating the cAMP-responsive element protein 1 (CREB1)/ALDH2/Wingless and int-1 (Wnt)/β-catenin axis [80]. Mechanistically, CREB1, activated by DSCR1-1, translocates into the nucleus and binds to the promoter of ALDH2, thus facilitating its gene transcription [80]. In turn, the activation of ALDH2 blocks the Wnt/β-catenin signaling pathway by disrupting the migration of β-catenin from the cytosol to the nucleus [80]. In addition, ALDH2 can inhibit apoptosis by suppressing the activation of the PI3K/Akt/nuclear factor kappa-B (NF-κB) signaling pathway, but the underlying mechanism remains unclear [81] (Fig. 3f).

#### Apoptotic cell clearance

In recent years, apoptosis and apoptotic cells have been confirmed to be of great significance for cellular homeostasis and human health [82]. Efferocytosis is programmed for the selective removal of apoptotic cells [82]. Dysfunctional efferocytosis can result in secondary necrosis of apoptotic cells and enlargement of the necrotic core, which in turn exacerbates the inflammatory response and cell death [82, 83]. Small GTPase Ras-related C3 botulinum toxin substrate (Rac) family members, such as Rac1/2, play crucial roles in this process by promoting actin and membrane rearrangement [83, 84]. Zhang et al. [85] reported that ALDH2 directly binds to Ras-related C3 botulinum toxin substrate 2 (Rac2) and prevents its degradation by the ubiquitination pathway, enhancing the clearance of apoptotic cells through efferocytosis (Fig. 3g).

In summary, ALDH2 functions in a sophisticated system to maintain cell viability through the regulation of apoptosis and clearance of apoptotic cells (Fig. 3). The combined control of apoptosis and apoptotic cell clearance via ALDH2 could be a novel target for human diseases and should be taken into consideration in future research.

### Necroptosis

Necroptosis is a caspase-independent, regulated form of necrosis whose activation process is dependent on kinase regulation. It has been confirmed that the induction of necroptosis priming occurs Mainly through 2 pathways. The initiation of necroptosis requires stimuli that are shared with apoptosis, such as the activation of death receptors or pattern recognition receptors (e.g., Z-DNA-binding protein 1 and toll-like receptor 3/4), along with the inhibition of CASP8 activity. The subsequent phosphorylation of a series of proteins leads to necrosome formation and the activation of mixed-lineage kinase domain-like protein, the necroptosis executor. In this process, receptor-interacting protein kinase 3 (RIPK3), a key component of the necrosome, phosphorylates mixed-lineage kinase domain-like protein, leading to its translocation to the plasma membrane, cell membrane perforation, and cell death [86].

ROS are critically involved in the initiation of necroptosis by facilitating RIPK1 autophosphorylation, contributing to its activation, which is essential for RIPK3 recruitment into the necrosome [87]. Researchers have revealed that ALDH2 is a negative regulator of ROS-dependent necroptosis and that its depletion results in ROS accumulation and accelerated high glucose-induced H9C2 cell necroptosis [88]. Consistently, ALDH2 activation has a noteworthy protective effect on myocardial necroptosis under exposure to alcohol through a reduction in the production of ROS [89]. During myocardial I/R injury, 4-HNE serves as the main contributor to promoting cardiomyocyte necroptosis by preventing the ubiquitin-mediated degradation of RIPK1, which could be mitigated through the activation of ALDH2 [90].

In summary, these findings indicate that ALDH2 regulates necroptosis through at least 2 mechanisms: inhibition of RIPK1 activation and promotion of RIPK1 degradation (Fig. 4). Additional work may be needed to determine whether ALDH2 can modulate the necroptotic cell death pathway through other necroptosis-associated proteins.

### Pyroptosis

Pyroptosis is a type of caspase-dependent inflammatory cell death characterized by plasma membrane permeabilization and the release of inflammatory cytokines, which are driven by gasdermin family proteins, mainly gasdermin-D (GSDMD) and gasdermin-E (GSDME). The initiation of pyroptosis requires the activation of inflammasome components, such as nucleotide-binding domain (NBD), leucine-rich repeat (LRR), and pyrin domain (PYD)-containing protein 3 (NLRP3), and absent in melanoma 2. During inflammasome activation, CASP1 can be cleaved, leading to the production of the N-terminal fragment of GSDMD. In contrast, GSDME-N is cleaved by CASP3. GSDMD-N or GSDME-N subsequently translocates to the plasma membrane and creates pores, resulting in cell death. In addition, CASP4/5 (known as CASP11 in mice) can be directly activated under LPS stimulation, which in turn cleaves GSDMD and mediates cell pyroptosis. This process is referred to as the noncanonical pyroptosis pathway [86].

ROS are essential activators of pyroptosis [91]. ALDH2 overexpression attenuates NLRP3 inflammasome activation and reduces pyroptosis occurrence by suppressing ROS production [92]. Lu et al. [93] reported that the failure of ALDH2 activation contributes to the accumulation of ROS and the inhibition of BCL2, resulting in the activation of CASP3-GSDME-mediated pyroptosis. Additionally, the inhibition of ALDH2 has been reported to promote the translocation of HDAC3 from the nucleus to the mitochondria. In turn, mitochondrial HDAC3 promotes hydroxyl-CoA dehydrogenase alpha subunit deacetylation, leading to activation of the ROS-mtDNA-NLRP3 axis to promote cell pyroptosis [94]** (**Fig. 4).

Collectively, these findings suggest that ALDH2 plays a critical inhibitory role in pyroptosis through the modulation of multiple signaling pathways. Notably, noncanonical pyroptosis shares similar activators with the canonical pathway; however, there are no data on its relationship with ALDH2. Therefore, further research is needed to determine whether ALDH2 regulates cell survival through the noncanonical pyroptotic pathway.

### Ferroptosis

Ferroptosis, another form of proinflammatory cell death, is mediated by iron-dependent Lipid peroxidation, which can attack lipid membranes and cause loss of cell integrity. The core mechanism of ferroptosis involves an imbalance between iron accumulation-mediated ROS Generation and the antioxidant defense system. Therefore, ferroptosis can be triggered in one way by inhibiting the activity of glutathione peroxidase 4 (GPX4), which is the main protective mechanism of lipid membranes against oxidative damage, or in another way by promoting ROS production [86].

In terms of mechanism, ALDH2 regulates ferroptosis through the following pathways (Fig. 4). First, ALDH2 dysfunction or deficiency contributes to ferroptosis by promoting ROS generation, as excessive ROS accumulation is critically involved in ferroptosis [95]. Second, transcriptional upregulation of acyl-CoA synthetase Long-chain family member 4 (ACSL4) and activation of ACSL4-dependent ferroptosis require the activation of specificity protein 1 (SP1), a classic transcription factor, which can be abrogated by ALDH2 [96]. Third, ovarian tumor deubiquitinase 5 (OTUD5), a functional cysteine protease with deubiquitinase activity, exerts noteworthy protective effects against ferroptosis by interacting with GPX4, suppressing its ubiquitination and enhancing its protein stability [97]. However, the loss of ALDH2 function caused by the *ALDH2*^rs671^ mutation leads to the accumulation of 4-HNE, which can disrupt the interaction between ovarian tumor deubiquitinase 5 and GPX4, resulting in massive ubiquitination and extensive degradation of GPX4 together with uncontrolled ferroptotic cell death [97]. Fourth, the activation of ALDH2 can promote the phosphorylation of extracellular signal-regulated kinase (ERK), which in turn facilitates the phosphorylation of CREB1 and contributes to the transcription of GPX4 and ALDH2, resulting in the inhibition of ferroptosis and the formation of a positive feed-forward loop for ALDH2 activation [98]. Fifth, ALDH2 can accelerate the production of lipid droplets to counteract ferroptosis [98].

Notably, accumulating evidence suggests that ferroptosis and autophagy are closely related. For example, GPX4 can be degraded via chaperone-mediated autophagy, triggering the initiation of ferroptosis [99]. Similarly, nuclear receptor coactivator 4 (NCOA4)-dependent ferritinophagy provides Fe^2+^ for ferroptosis activation [100]. As a key regulator of autophagy, however, there is no direct evidence showing that ALDH2 can regulate ferroptosis via the autophagic machinery. Thus, further study is needed to determine the role of ALDH2 in modulating ferroptosis through the autophagic pathway.

### NETosis

NETosis is a regulated form of neutrophil cell death characterized by the release of neutrophil extracellular traps (NETs) in response to infection or injury. The activation of NETosis can not only prevent bacteria and other pathogens from spreading by capturing or killing them but also promote the release of damage-associated molecular patterns, thus contributing to the development of various diseases, such as sepsis, cardiovascular diseases, and cancer. Multiple steps in the process of NETosis have been described. First, nicotinamide adenine dinucleotide phosphate (NADPH) oxidase promotes ROS generation, which in turn facilitates the release and translocation of granular enzymes and peptides from the cytosol to the nucleus. Subsequently, the granular enzymes and peptides trigger the citrullination of histone H3, leading to chromatin decondensation and NETosis. Notably, peptidyl arginine deiminase 4 (PAD4) is critically involved in this process by promoting histone citrullination [86]. However, the precise molecular mechanisms of NETosis have not been fully elucidated and require further mechanistic investigation.

Recently, it was reported that ALDH2 can bind to the E3 ubiquitin Ligase C-terminus of Heat shock protein 70 (HSC70)-interacting protein (CHIP) to increase the activity of the C-terminus of Hsc70-interacting protein and promote the ubiquitination of PAD4, contributing to the massive degradation of PAD4 and inhibition of NETosis [101]. In contrast, loss of ALDH2 function significantly aggravates necrotic cell death and cytotoxic activity in endothelial cells during sepsis, whereas treatment with Alda-1 can mitigate these effects [101]. Moreover, Yang et al. [102] demonstrated that ALDH2 deficiency induces NADPH oxidase 2-mediated NETosis in an ROS-dependent manner. Mechanistically, ALDH2 depletion activates the ER stress/microsomal glutathione S-transferase 2 (MGST2)/leukotriene C4 (LTC4) pathway, which in turn triggers NADPH oxidase 2 (NOX2)-induced ROS accumulation and NETosis. Notably, pharmacological inhibition of the ER stress/MGST2/LTC4 pathway through treatment with the LTC4 receptor antagonist pranlukast significantly alleviates NETosis, suggesting that LTC4 might be a potential target to regulate NETosis, particularly for individuals with *ALDH2* gene mutations that largely compromise the function of ALDH2 [102] **(**Fig. 4).

Taken together, these findings suggest that the functional aberration of ALDH2 is critically involved in activating NETosis and accelerating neutrophil death. In contrast to the above ‘suicidal’ NETosis, a ‘vital’ form of NETosis has recently been identified in which neutrophils remain viable after releasing extracellular traps [103]. Further studies are needed to determine whether ALDH2 selectively regulates ‘vital’ versus ‘suicidal’ NETosis to control neutrophil fate.

## ALDH2 regulates autophagy and cell death in human diseases

Under normal conditions, an organism removes unnecessary cellular substances or cells, either by initiating autophagy or activating cell death, thereby maintaining homeostasis [1]. However, aberrant autophagy and cell death can result in lethal or uncontrolled inflammatory responses, which are the main causes of many human diseases [43]. Impaired ALDH2 activity caused by *ALDH2* gene mutation or ALDH2 deficiency is intimately associated with dysfunctional autophagy and RCD. Therefore, targeting ALDH2 is an effective approach for maintaining human health. Here, we discuss the pathological role and therapeutic potential of ALDH2, autophagy, and cell death in human diseases.

### Cardiovascular diseases

**Fig. 5:**
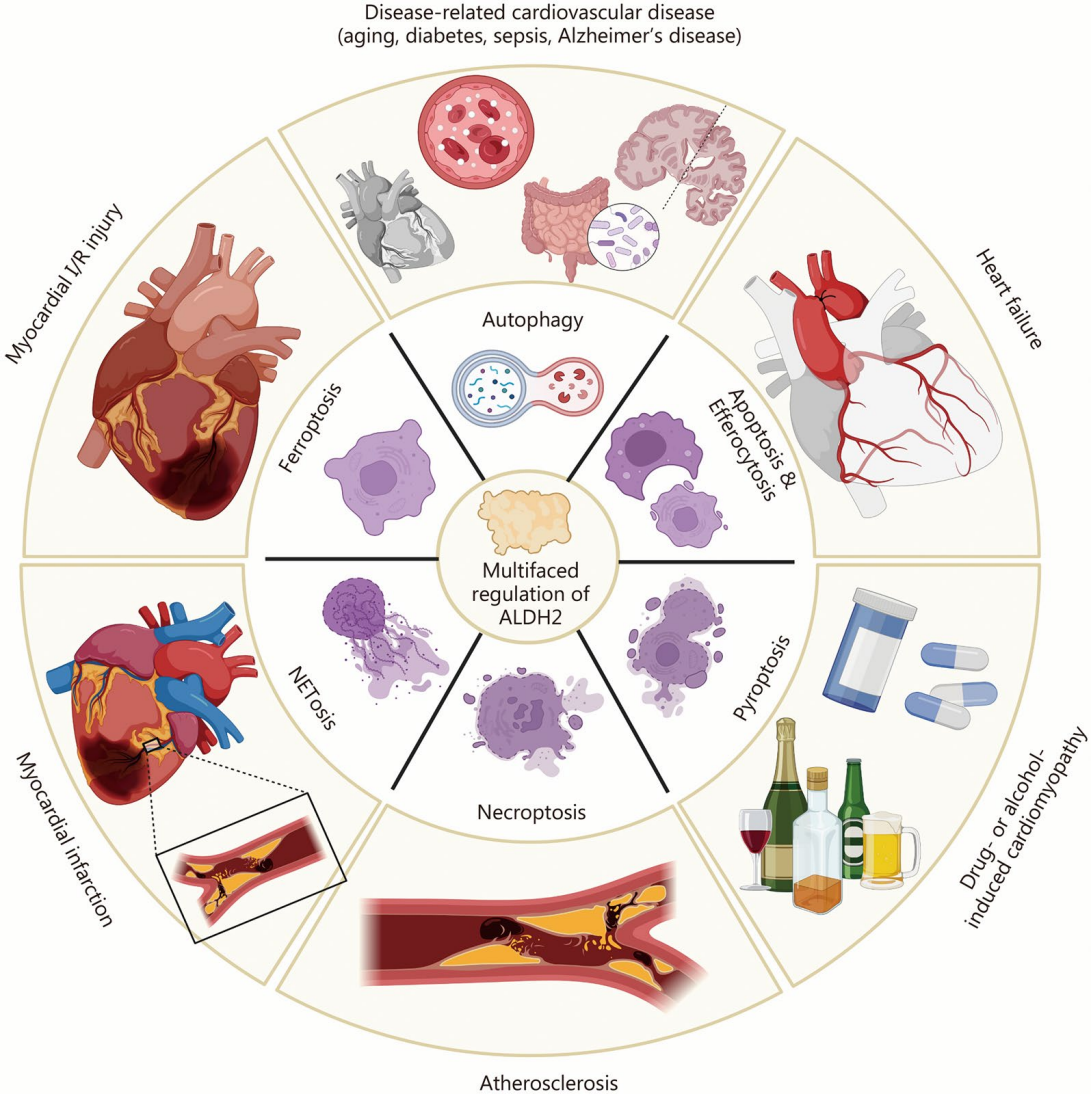
ALDH2 regulates autophagy and cell death in cardiovascular diseases. Summarizing the type of cell death and autophagy in cardiovascular diseases. ALDH2 aldehyde dehydrogenase 2, I/R ischemia/reperfusion

In recent years, it has become increasingly clear that the defective activation of ALDH2 is the main reason for the occurrence and development of various cardiovascular diseases [6]. Moreover, emerging evidence has shown that aberrant activation of autophagy and cell death are critically involved in these diseases and are intimately associated with dysfunctional ALDH2. Here, we discuss the clinical implications and therapeutic potential of ALDH2-related autophagy and cell death in cardiovascular diseases (Fig. 5).

#### Myocardial I/R injury

Myocardial I/R injury is characterized by a large burst of cardiomyocyte death [104]. RCD plays a crucial role in this process, and inhibition of RCD is important for delaying myocardial I/R progression [104]. Recently, 4-HNE was reported to aggravate myocardial I/R injury by promoting cardiomyocyte necroptosis, and this effect could be abrogated by facilitating the activation of ALDH2 [90]. Liu et al. [97] revealed that during myocardial I/R injury, the accumulation of Massive amounts of 4-HNE exacerbates myocardial ferroptosis and uncontrolled inflammatory responses owing to ALDH2 dysfunction in heart tissue. The administration of Alda-1 significantly improved cardiac pathology caused by ischemia injury by promoting mitochondrial biogenesis and attenuating myocardial apoptosis [71]. Elevated NETosis was detected in neutrophils from myocardial I/R patients with the *ALDH2*^rs671^ mutation and ischemic myocardium from *ALDH2* knockout mice. The targeted inhibition of NETosis or overexpression of ALDH2 could significantly inhibit NETosis, promote angiogenesis, and ameliorate cardiac function [102]. Notably, an imbalance between mitochondrial fission and fusion is also a main factor in the pathogenesis and progression of myocardial I/R injury, which can contribute to energy disorders and cell death [61]. ALDH2 was confirmed to inhibit I/R-induced excessive mitochondrial fission and maintain cardiomyocyte survival, thus ameliorating hypoxia- and reoxygenation-induced cardiomyocyte injury [105]. Tan et al. [68] reported that during diabetic myocardial I/R injury, increased ROS levels disrupt the balance between mitochondrial fusion and fission, thereby exacerbating damage, which can be reversed by ALDH2 activation. In addition, the activation of ALDH2 can inhibit cell death and alleviate myocardial I/R injury by enhancing the effects of other therapeutic pathways; the induction of ALDH2 enhances the therapeutic effects of mitochondrial transplantation for myocardial I/R injury [67]. Enhanced ALDH2 activity is one major reason for the cardioprotective effect of cardiac hypertrophic preconditioning on improving myocardial I/R injury [76]. Shexiang Baoxin pill, a well-known Chinese medicine, can alleviate ischemic injury in an ALDH2-dependent manner [106]. Intriguingly, Ma et al. [44] reported that different ALDH2-mediated autophagy regulatory effects have the same effects on the development of myocardial I/R injury. Mechanistically, during ischemia, increased autophagy activated by ALDH2 is the main reason for the maintenance of cell viability in response to hypoxia stimulation. Conversely, the inhibition of autophagy caused by ALDH2 activation has a noteworthy protective effect against heart injury by suppressing autophagy during the reperfusion stage [44]. Similarly, excessive mitophagy is induced during myocardial I/R injury to aggravate cardiomyocyte death and promote cardiac dysfunction. The activation of ALDH2 exerts a cardioprotective effect in this process by regulating PINK/PRKN expression and suppressing excessive mitophagy [55]. These studies demonstrate that the role of ALDH2 in regulating autophagy during the progression of myocardial I/R injury is disparate and specific. Further understanding the regulatory mechanisms of ALDH2 in autophagy might improve the prevention and treatment of myocardial I/R injury.

#### Atherosclerosis

Atherosclerosis is a chronic inflammatory vascular disease, and various factors, such as a persistent inflammatory response, macrophage foam cell formation, and endothelial cell injury, are closely related to the occurrence and development of atherosclerosis [107]. Yang et al. [74] reported that ALDH2 alleviates ER stress-mediated smooth muscle cell apoptosis, thereby slowing the progression of atherosclerosis. miR-193b-3p can aggravate endothelial cell injury in atherosclerosis by downregulating ALDH2 expression and exacerbating ER stress-related apoptosis [75]. It has been reported that macrophage expression of ALDH2 is conducive to limiting the persistent inflammation during atherosclerosis through the inhibition of Rac2 degradation and the promotion of macrophage efferocytosis [85]. The inhibition of ALDH2 expression in macrophages can inhibit their capacity for efferocytosis and promote atherosclerosis, indicating that ALDH2 deficiency promotes atherosclerosis [85]. However, macrophage ALDH2 expression can also contribute to atherosclerosis, as AMPK-phosphorylated ALDH2 was confirmed to result in impaired lysosomal function, blocked autophagy, increased lipid deposition, and accelerated foam cell formation. Interfering with macrophage ALDH2 expression can significantly improve atherosclerosis, suggesting that ALDH2 deficiency plays a protective role in atherosclerosis [42]. Interestingly, *ALDH2*^rs671^ in macrophages can accelerate Rac2 degradation and promote the AMPK-mediated phosphorylation of ALDH2 to aggravate atherosclerosis [42, 85]. In summary, these findings indicate that the role of ALDH2 deficiency and ALDH2 dysfunction in atherosclerosis is distinct and closely related to the type of tissue and cell. Therefore, targeting ALDH2 expression in a tissue-specific manner or correcting ALDH2 functional mutation in a cell-specific manner might be a therapeutic option to balance the dual role of ALDH2 in atherosclerosis.

#### Heart failure (HF)

HF, the end stage of various cardiovascular diseases, is among the major contributors to mortality in many countries [108, 109]. The underlying mechanisms that account for the progression of HF are not completely understood; thus, the treatment for HF has yet to be improved. It was reported that alpha-lipoic acid, a well-known antioxidant, rescued pressure overload-induced heart injury in an HF model through ALDH2-dependent FUNDC1-mediated mitophagy [51]. Elevated 4-HNE showed a serious adverse effect on cardiomyocytes during HF by promoting apoptosis, and this effect was mitigated by upregulating ALDH2 expression [79]. Failed activation of ALDH2 and inhibition of mitophagy are closely related to the pathogenesis of HF, as decreased ALDH2 activity, defective mitophagy, and increased cardiomyocyte apoptosis were observed in HF rats [110]. Treatment with Alda-1 in HF rats overtly ameliorated injury by promoting mitophagy and attenuating cardiomyocyte apoptosis [110]. In summary, these results demonstrate that maintaining functional ALDH2 to regulate cell death or autophagy might be a high priority for the treatment of HF.

#### Drug- or alcohol-induced cardiomyopathy

Deficient autophagy and uncontrolled cell death contribute to lethal inflammation and cardiac dysfunction, which are both major causes of the progression of drug- or alcohol-induced cardiomyopathy [111, 112]. A growing body of evidence suggests that ALDH2 is also involved in protecting against drug- or alcohol-induced cardiotoxicity. For example, overactivation of autophagy caused by ALDH2 inhibition is closely associated with the pathogenesis of doxorubicin-induced myocardial dysfunction [113]. Gao et al. [114] reported that the activation of ALDH2 alleviates doxorubicin-induced cardiotoxicity by inhibiting oxidative stress and reducing myocardial apoptosis. In addition, ALDH2 deficiency can result in cardiac dysfunction induced by alcohol consumption by promoting myocardial apoptosis and necroptosis [89].

#### Disease-related cardiovascular disease

Cardiac dysfunction is a common complication of many diseases, and its presence accelerates and aggravates disease development [115]. Consistently, ALDH2 plays an indispensable role in disease-related cardiac dysfunction. Taking diabetic cardiomyopathy as an example, ALDH2 is extensively activated to detoxify 4-HNE and maintain cardiomyocyte survival, while impaired ALDH2 activity results in massive cardiomyocyte death, lethal inflammation, and severe cardiac dysfunction [66]. Zhang et al. [113] found that ALDH2 plays a critical role in inhibiting the progression of diabetic cardiomyocyte by effectively removing damaged mitochondria through PRKN-dependent mitophagy. During high glucose-induced cardiac injury, the expression of the necroptosis regulator RIPK3 and the pyroptosis regulator NLRP3 is increased, and treatment with Alda-1 can alleviate cardiac injury by suppressing cardiomyocyte pyroptosis and necroptosis, respectively [92, 116]. Necroptosis is also an important contributor to the acceleration of cardiomyocyte fibrosis during diabetic cardiomyopathy, and treatment with Alda-1 can alleviate cardiomyocyte necroptosis to delay myocardial fibrosis [88]. Under exposure to high glucose, the expression and activity of ALDH2 in cardiac fibroblasts can be suppressed, which in turn induces cardiac fibroblast apoptosis and accelerates myocardial fibrosis [117]. Notably, emerging studies suggest a high incidence of cardiovascular events in AD, but neither the exact pathogenesis nor an effective therapeutic target is available [96, 115]. Recently, researchers found that the overexpression of ALDH2 in AD model mice rescues cardiac contractile dysfunction through the suppression of ACSL4-mediated ferroptosis [96]. Low levels of serum melatonin and inhibition of ALDH2 activity were observed in AD patients and model mice, along with evident dysfunction of cardiac function. Treatment with melatonin markedly ameliorated defective cardiac function through enhancing mitophagy and inhibiting cardiac apoptosis in an ALDH2-dependent manner [50]. These findings suggest that ALDH2 might be a novel therapeutic target for AD-related cardiac dysfunction. In addition, septic cardiomyopathy is among the main complications of sepsis and is an important factor contributing to the high mortality rate of sepsis [118]. Zhang et al. [94] found that ALDH2 is activated in the cardiomyocytes of septic mice to inhibit pyroptosis, thus ameliorating cardiac dysfunction. ALDH2 was also reported to be involved in the default response to endotoxic stimulation, as its ablation results in aberrant activation of the STING pathway and extensive cardiomyocyte apoptosis [78]. Notably, ALDH2 has been demonstrated to have a damaging effect on aging-related heart disease. During the aging process, ALDH2 aggravates the aging-induced inhibition of autophagy, which in turn accelerates myocardial remodeling and contractile dysfunction [45].

#### Myocardial infarction (MI)

MI is a life-threatening coronary-associated pathology characterized by sudden cardiac death [119]. Calycosin was found to have a cardioprotective effect in an MI model by protecting against oxidative stress-induced cardiomyocyte apoptosis in an ALDH2-dependent manner, and ALDH2 activity is negatively correlated with the MI area [63].

Collectively, these data indicate that dysfunctional ALDH2 contributes to the pathogenesis of various cardiovascular diseases and could be a promising therapeutic target for their treatment.

### Brain disorders

The main cause of brain disorders is the gradual loss of brain structure and function caused by the progressive degeneration and/or death of nerve cells [120]. Multiple strategies that aim to limit inflammation and control neuronal cell death are assumed to be effective therapies for patients with brain diseases. A recent study revealed that ALDH2 deficiency increases susceptibility to septic brain injury by promoting NLRP3 activation, increasing cell pyroptosis, and aggravating lethal inflammation [121]. ALDH2 plays a crucial role in the Takeda G protein-coupled receptor 5-mediated protective effect on neurons in rats with subarachnoid hemorrhage. Ablation of ALDH2 expression significantly accelerates neuronal apoptosis and abates the neuroprotective effects of Takeda G protein-coupled receptor 5 [122]. In a cerebral I/R injury model, ALDH2 suppressed the activation of multiple pathways, including apoptosis, pyroptosis, ferroptosis, and autophagy, to maintain cell viability [123]. ALDH2 has also been confirmed to protect the brain against I/R injury induced by cardiac arrest and resuscitation in a swine model, in which the protective effect was associated with the suppression of pyroptosis [124]. In addition to acute brain injury, the overexpression of ALDH2 can improve cognitive ability in AD model mice by alleviating amyloid β-peptide accumulation-induced apoptosis [62]. Decreased expression and activity of ALDH2 were found in a rat model of depression, together with Massive accumulation of 4-HNE and extensive apoptosis in the hippocampus and prefrontal cortex [125]. Another study demonstrated that histidine triad nucleotide-binding protein 1 is involved in delaying the progression of depression by alleviating oxidative stress and apoptosis in the prefrontal cortex through the protein kinase C ε (PKCε)/ALDH2/4-HNE pathway [126]. Yan et al. [58] reported that ethanol treatment induces excessive mitophagy and cytotoxicity in N2a cells and that these effects can be significantly inhibited by promoting ALDH2 activity. Additionally, ALDH2 activation can alleviate alcohol-induced brain injury by inhibiting enterocyte apoptosis, improving gut leakiness, and reducing endotoxin release, suggesting that the regulation of the gut–brain axis through ALDH2 might be a potential target for alcohol-mediated gut and brain injury [127].

**Fig. 6:**
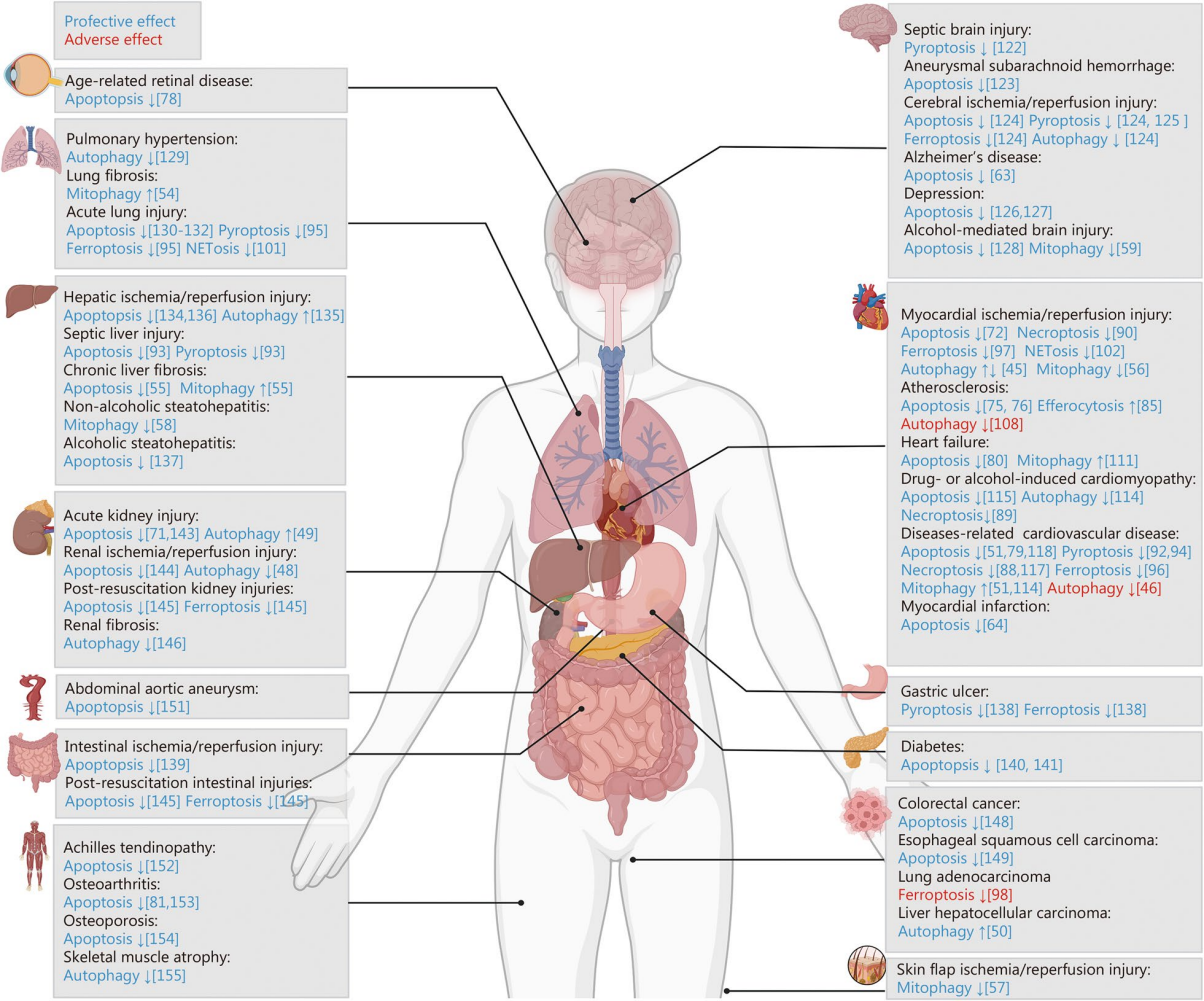
The effect of ALDH2-regulated autophagy and cell death in human diseases. Functional ALDH2-regulated autophagy and cell death are involved in various human diseases, with a protective effect or an adverse effect. ALDH2 aldehyde dehydrogenase 2

In summary, the above data demonstrate that ALDH2 dysfunction is a key driver of neurodegenerative processes, mediating both neuronal survival deficits and pathological inflammation. Therefore, modulating ALDH2 could be a novel interventional strategy for treating neurodegenerative disorders (Fig. 6).

### Respiratory system diseases

Emerging evidence has demonstrated that ALDH2 plays a broad role in regulating cell death sensitivity and autophagy to delay the development of respiratory system diseases. First, increased autophagy caused by ALDH2 deficiency was confirmed to promote the migration and proliferation of pulmonary arterial smooth muscle cells, accelerating the pathological progression of pulmonary hypertension [128]. Decreased ALDH2 expression was noted in patients with idiopathic pulmonary fibrosis and in mice with bleomycin-induced pulmonary fibrosis, along with evident impairment of mitophagy, which is positively correlated with fibroblast activation and abnormal extracellular matrix accumulation [53]. Second, of 4-HNE accumulation, massive apoptosis, and extensive inflammation have been observed in lung tissue during embolism-induced acute lung injury, and this lung damage can be alleviated by the administration of Alda-1 [81]. The activation of ALDH2 inhibits apoptosis and downregulates the excessive inflammatory response to improve acute lung injury after cardiac arrest and cardiopulmonary resuscitation in swine [129]. Similarly, pharmacological activation of ALDH2 can rescue heatstroke-induced acute lung injury by alleviating oxidative stress and decreasing endothelial cell apoptosis [130]. Third, it has been reported that ALDH2 plays a protective role in sepsis-induced lung injury by inhibiting pyroptosis and ferroptosis [95]. Fourth, Xu et al. [101] reported that during sepsis, patients with the *ALDH2*^rs671^ mutation are susceptible to developing septic acute respiratory distress syndrome, which is closely related to uncontrolled NETosis.

In summary, these results demonstrate that ALDH2 plays a distinct and specific role in respiratory system diseases. A deeper understanding of the regulatory mechanisms of ALDH2 could lead to improved strategies for preventing and treating respiratory system diseases (Fig. 6).

### Digestive system diseases

Digestive system diseases have become a growing global burden because of insufficient therapeutic measures [131]. Recently, emerging evidence has confirmed that ALDH2 might be a new potential target for treating related diseases. Alda-1 treatment reduces the sensitivity of hepatocytes to apoptosis induced by I/R injury by increasing ALDH2 activity and inhibiting oxidative stress [132]. Liu et al. [133] reported that the inhibition of autophagy is detrimental to hepatic I/R injury, whereas pretreatment with Alda-1 can rescue the liver of rats from I/R injury by promoting autophagy. Researchers have also reported that increased ALDH2 activity can reduce the high vulnerability of liver grafts by attenuating I/R-induced apoptosis [134]. During the development of sepsis, increased hepatocyte apoptosis and GSDME-mediated pyroptosis were confirmed to be the major causes of septic liver injury, which could be alleviated by promoting ALDH2 activity [93]. The induction of PRKN-mediated mitophagy in an ALDH2-dependent manner was shown to improve the classic toxicity of carbon tetrachloride-induced chronic liver fibrosis by inhibiting ROS production and reducing apoptosis [54]. Conversely, the activation of ALDH2 can suppress excessive PRKN-mediated mitophagy and preserve mitochondrial function to delay the progression of vinyl chloride-induced nonalcoholic steatohepatitis [57]. Pharmacological activation of ALDH2 improves alcoholic steatohepatitis by inhibiting ER stress and apoptosis [135]. With respect to gastrointestinal disease, increased pyroptosis and ferroptosis caused by ALDH2 deficiency have been confirmed to be closely associated with the pathogenesis of gastric ulcer injury [136]. Zhu et al. [137] reported that increased ALDH2 activity alleviates I/R-induced intestinal injury through the clearance of toxic aldehydes and the suppression of apoptosis. Notably, the abnormal accumulation of reactive aldehydes, such as methylglyoxal and 4-HNE, contributes to diabetes. Specifically, 4-HNE is involved in lethal inflammatory responses and activation of the cell death pathway [6]. ALDH2 is an important limiting factor for diabetes, and its deletion significantly exacerbates pancreatic beta cell apoptosis in response to glucolipotoxicity [138]. Additionally, a higher risk of diabetes is associated with prolonged exposure to chemotherapeutic agents, such as doxorubicin. Researchers have demonstrated that ALDH2 deficiency aggravates the cytotoxic activity of pancreatic beta cells in response to doxorubicin, while Alda-1 treatment exerts significant cytoprotective effects by inhibiting apoptosis [139]. Overall, these data demonstrate that ALDH2 dysfunction drives the pathogenesis of digestive system diseases, positioning it as a viable therapeutic target (Fig. 6).

### Kidney diseases

Kidney diseases have become major causes of high morbidity and mortality rates across the globe [140]. However, rapid tests, accurate diagnosis, and available interventions for kidney diseases have yet to be realized. Moreover, markers for monitoring the occurrence or deterioration of kidney diseases are lacking. In a recent study, researchers found that ALDH2 expression is significantly decreased in cisplatin- or maleic acid-induced acute kidney injury (AKI) models, along with mitochondrial dysfunction and tubular epithelial cell apoptosis [70]. ALDH2 activation restores mitochondrial membrane potential and inhibits apoptosis by promoting peroxisome proliferator-activated receptor gamma coactivator-1 α-dependent mitochondrial biogenesis [70]. ALDH2 was also confirmed to regulate cellular ROS levels to alleviate tubular epithelial cell apoptosis and renal injury by promoting autophagy through the BECN1 pathway during contrast-induced AKI [48]. Notably, ALDH2 expression was found to be a default response to endotoxic stimulation, as its deficiency resulted in the accumulation of 4-HNE, suppression of the mitogen-activated protein kinase (MAPK) pathway, and the exacerbation of tubular epithelial cell apoptosis in LPS-induced AKI [141]. With respect to renal I/R injury, Chen et al. [142] reported that ALDH2 plays a critical role in improving renal I/R injury by reducing tubular epithelial cell apoptosis. During renal transplantation, hypothermic machine perfusion has a noteworthy protective effect against I/R-induced kidney injury by inhibiting excessive autophagy through regulation of the Akt/mTOR autophagic pathway in an ALDH2-dependent manner [47]. Yu et al. [143] demonstrated that ALDH2 can be an effective therapeutic target for improving postresuscitation renal and intestinal injury through the suppression of apoptosis and ferroptosis. In addition to its role in acute kidney diseases, ALDH2 is also critically involved in chronic kidney diseases. Astragaloside IV, a natural saponin extracted from Astragali radix, can upregulate ALDH2 expression, which in turn inhibits autophagy, mitigates epithelial–mesenchymal transition and G2/M arrest, and suppresses the development of renal fibrosis [144].

Taken together, these findings suggest that ALDH2 dysfunction not only has potential as a dual-purpose biomarker (diagnostic and prognostic) for kidney diseases but can also act as a promising therapeutic target to prevent and treat kidney diseases (Fig. 6).

### Cancer

Autophagy and cell death promote the occurrence and development of cancer priming in the following 2 Main ways: 1) dysfunctional autophagy and aberrant cell death can result in persistent inflammation and tissue damage, which increase the risk of tumorigenesis; and 2) cancer cells can hijack autophagy or inhibit the cell death pathway to maintain cell viability and facilitate tumor progression [145]. For example, the inhibition of ALDH2 is involved in colorectal cancer progression through the promotion of DNA damage and apoptosis [146]. Cells with high expression of CD44 (CD44H cells) are recognized as cancer stem cells involved in the development of esophageal squamous cell carcinoma (ESCC), which is characterized by high proliferation and drug resistance [147]. Recently, researchers reported that under alcohol exposure, the *ALDH2*^rs671^ mutation exacerbates DNA damage and apoptosis in non-CD44H cells, which contributes to the enrichment of CD44H cells and accelerates ESCC development [147]. ALDH2 deficiency or the *ALDH2*^rs671^ mutation can increase sensitivity to platinum-based chemotherapy in lung adenocarcinoma by increasing ferroptosis in lung adenocarcinoma tissues [98]. Additionally, during the development of liver hepatocellular carcinoma, increased autophagy mediated by ALDH2 activation can promote the infiltration of T cells in tumors to limit cancer cell immune escape and suppress tumor growth [49].

Notably, while these studies demonstrate promising findings, they are currently limited to investigating the role of ALDH2 in a single tissue type. Further research is needed to elucidate the effects and mechanisms of ALDH2 in different tissues during the progression of cancer (Fig. 6).

### Other human diseases

Random skin flap transplantation is a common method for repairing tissue defects, such as burns or trauma. However, during this process, I/R injury and inflammation are major causes of aggravated flap necrosis, leading to poor outcomes [148]. Recently, researchers reported that Alda-1 treatment effectively improves random skin flap viability by inhibiting the activation of PINK1/PRKN-dependent mitophagy [56]. In abdominal aortic aneurysm (AAA), ALDH2 inhibits aneurysm formation by inhibiting ROS production, reducing vascular inflammation, and attenuating vascular smooth muscle cell apoptosis, suggesting that ALDH2 can serve as a novel treatment option for AAA [149]. With respect to age-related retinal disease, ALDH2 overexpression in aging mice can alleviate oxidative stress-induced retinal cell apoptosis [77]. In addition, ALDH2 strongly contributes to protection against musculoskeletal diseases through the regulation of autophagic machinery or cell death pathways. In a recent report, researchers revealed the beneficial effects of ALDH2 activation on Achilles tendinopathy through the inhibition of oxidative stress and apoptosis [150]. ALDH2 is involved in slowing the progression of osteoarthritis by inhibiting oxidative stress, inflammation, and apoptosis through the regulation of aquaporin-4 expression [151]. The DSCR1-1/CREB1/ALDH2/Wnt/β-catenin axis has been demonstrated to be involved in the etiology of osteoarthritis. Mechanistically, DSCR1-1 upregulates ALDH2 expression in a CREB1-dependent manner [80]. ALDH2 activation subsequently blocks the Wnt/β-catenin signaling pathway, inhibiting chondrocyte apoptosis and improving osteoarthritis [80]. Aldehyde stress caused by the *ALDH2*^rs671^ mutation can accelerate osteoporosis progression by inducing apoptosis and suppressing differentiation and proliferation in osteoblasts [152]. Zhang et al. [153] reported that exhaustive exercise induces skeletal muscle atrophy by reducing ALDH2 levels and promoting excessive autophagy. Skeletal muscle-specific ALDH2 transgenic mice show opposite outcomes in response to similar stimuli, suggesting a protective mechanism against skeletal muscle atrophy (Fig. 6).

## Therapeutic strategies targeting ALDH2

These data reveal that ALDH2 deficiency or dysfunction plays a crucial role in a wide range of human diseases. There is an urgent need to discover new therapies that can solve these problems.

### ALDH2 modification

Modifications of ALDH2 have been confirmed not only to regulate ALDH2 expression but also to significantly affect ALDH2 activity, which has profound implications for human diseases. Posttranslational modification (PTM) refers to the regulation of protein activity, localization, and interactions with cellular molecules by the addition or removal of specific groups on the amino acid residues of target proteins [154, 155]. Common PTMs include phosphorylation, acetylation, methylation, lipidation, and ubiquitination, which greatly increase the diversity of proteome research [154, 155]. In recent years, a series of PTMs, such as phosphorylation, ubiquitination, and acetylation, have been confirmed to significantly affect the functional activity of ALDH2, which is strongly associated with human diseases [156–159]. Therefore, intensive investigation of ALDH2 PTMs is important for understanding the regulatory mechanisms of ALDH2 activity, identifying drug targets, and screening clinical markers.

#### Phosphorylation

Phosphorylation is the process of adding a phosphate group to an intermediate metabolite or to a protein and is responsible for protein activity regulation, energy metabolism, and signal transduction [154]. ALDH2 can be phosphorylated by PKCε at Thr185, Thr412, and Ser279, which is crucial for maintaining ALDH2 enzymatic activity [156]. The phosphorylation of ALDH2 mediated by PKCε has a noteworthy protective effect against myocardial I/R, MI, and AD-induced cardiac dysfunction [50, 156, 157]. Similarly, researchers have reported that activation of the PI3K signaling pathway improves myocardial I/R injury in female rats by increasing the phosphorylation and enhancing the enzymatic activity of ALDH2, but the exact site of ALDH2 phosphorylation in this process remains unclear [158]. Intriguingly, AMPK activation caused by the *ALDH2*^rs671^ mutation can phosphorylate ALDH2 at Thr356 and Tyr148, contributing to the nuclear translocation of ALDH2 and the transcriptional regulation of *ATP6V0E2* expression [42]. Notably, phosphorylation mediated by JNK at Ser residues of ALDH2 (Ser74, Ser273, and Ser463) leads to a conformational change in its substrate-binding domain, which in turn inhibits ALDH2 activity [159].

#### Acetylation/deacetylation

Reversible protein acetylation is an important regulatory mechanism for regulating protein function, such as gene transcription and amino acid catabolism [160]. Mitochondria are the Major sites of protein acetylation, as most mitochondrially localized proteins contain lysine acetylation sites. Sirtuin 3 (SIRT3) is localized mainly in the mitochondrial matrix and functions as a key enzyme involved in the process of deacetylation [160, 161]. It has been reported that the induction of ALDH2 acetylation at lysine369 can perturb NAD^+^ cofactor binding and inhibit ALDH2 activity, which is partly reversed by SIRT3-mediated deacetylation [162]. In addition to suppressing ALDH2 activity, Xue et al. [163] demonstrated that SIRT3 inactivation leads to a significantly increased level of ALDH2 acetylation but, paradoxically, promotes ALDH2 activity. However, how acetylation affects ALDH2 activity in this process is unclear and remains to be further elucidated. Notably, activated ALDH2 can also increase SIRT3 activity, suggesting that there might be a feedback mechanism, but the details of this mechanism need further investigation.

#### Others

S-nitrosylation is a selective covalent posttranslational modification that adds a nitroso group to the active thiol group of cysteine in the form of S-nitrosothiol and modulates multiple cellular processes [164]. ALDH2 can be S-nitrosylated at cysteine 302, triggering a significant reduction in its catalytic activity [165]. Ubiquitination is characterized by the selective degradation of tagged proteins through the proteasomal pathway and has been confirmed as a key determinant of protein fate [154, 155]. During hepatitis B virus infection, the hepatitis B virus X protein can interact with mitochondrial ALDH2 to promote its ubiquitination and degradation [166]. Another study demonstrated that upregulating SIRT3 expression diminishes ALDH2 lactylation at lysine 52 and promotes its interaction with PHB2, facilitating mitophagy, improving mitochondrial dysfunction, and delaying the progression of AKI [52]. Additionally, ALDH2 can be modified by succinylation at K385, leading to a notable decrease in ALDH2 activity and aggravation of acetaminophen-induced liver injury in a mouse model [167]. Conversely, SIRT5 activation can reverse this injury through the desuccinylation of ALDH2 and maintenance of its enzymatic activity [167].

Although significant advances have been made in ALDH2 modification, the regulatory effects of some PTMs, such as SUMOylation, on ALDH2 activity are still ambiguous (Table 2) [42, 52, 156, 158, 159, 162, 163, 165–167]. Thus, more work is needed to investigate their role in modulating ALDH2 activity, which could provide reliable targets for clinical drug development and treatment.

**Table 2:** The function of post-translational modification of ALDH2

Modification	Site	Regulator	Functional effect	References
Phosphorylation	Thr185, Thr412, Ser279	PKCε	Maintain ALDH2 enzymatic activity	[156]
Phosphorylation	Unknown	PI3K pathway	Enhance ALDH2 enzymatic activity	[158]
Phosphorylation	Thr356, Tyr148	AMPK	Promote ALHD2 nuclear translocation and regulate *ATP6V0E2* expression	[42]
Phosphorylation	Ser74, Ser273, Ser463	JNK pathway	Inhibit ALDH2 enzymatic activity	[159]
Acetylation	Unknown	SIRT3	Promote ALDH2 enzymatic activity	[163]
Deacetylation	Lys369	SIRT3	Maintain ALDH2 enzymatic activity	[162]
S-nitrosylation	Cys302	Unknown	Suppress ALDH2 enzymatic activity	[165]
Ubiquitination	Unknown	HBV X protein	Promote ALDH2 degradation	[166]
Delactylation	Lys52	SIRT3	Promote ALDH2 interaction with PHB2 to inhibit PHB2 degradation	[52]
Desuccinylation	K385	SIRT5	Maintain ALDH2 enzymatic activity	[167]

*ALDH2* aldehyde dehydrogenase 2, *AMPK* AMP-activated protein kinase, *ATP6V0E2* ATPase H^+^ transporting V0 subunit E2, *HBV* hepatitis B virus, *JNK* c-Jun N-terminal kinase, *SIRT3* sirtuin 3, *PHB2* prohibitin 2, *PI3K* phosphatidylinositol 3-kinase, *PKCε* protein kinase C ε

### ALDH2 modulators

In addition to regulating ALDH2 activity through PTMs, various ALDH2 modulators have been used in cellular or animal models to treat human diseases. The details of these modulators are summarized in Table 3 [45, 51, 66, 74, 98, 168–185].

**Table 3:** Potential modulators of ALDH2

Drugs	Target (ALDH2)	Effect/Clinical trial phase	diseases	References/Trial ID
Activator				
Alda-1	Activity	Improve the corresponding injury and delay the development of the disease	Various human diseases	[45]
	Activity	Aggravate aging-induced cardiac dysfunction	Cardiac aging	[45]
	Activity	Enhance platinum chemoresistance in LUAD tissue	LUAD	[98]
Alda-44	Activity	Improve cardiac dysfunction	Myocardial I/R injury	[168]
α-lipoic acid	Activity	Improve cardiac dysfunction	Myocardial I/R injury	[169]
	Activity	Improve pressure overload-induced heart failure	Heart failure	[51]
AD-9308	Activity	Improve left ventricular diastolic and systolic functions	Diabetic cardiomyopathy	[66]
	Activity	Improve obesity-associated metabolic disorders	Obesity	[170]
	Activity	Improve miRNA maturation and cardiac function/remodelling	Heart failure	[171]
Magnolol	Activity	Inhibit cardiac fibroblast proliferation and collagen synthesis	Cardiac fibrosis	[172]
Flurbiprofen	Activity	Attenuate high-fat diet-induced obesity	Obesity	[173]
Compound C6	Activity	Reduce infarct size by about 70%	Ischaemic stroke	[174]
Baicalin	Activity and expression	Improve cardiac dysfunction	Myocardial I/R injury	[175]
Dihydromyricetin	Activity	Alleviate neuropathic pain	Neuropathic pain	[176]
Hovenia dulcis	Activity and expression	Detoxify alcoholism	Hangovers	[177]
Shexiang Baoxin	Expression	Improve cardiac dysfunction	Ischemic injury	[106]
Antrodia Cinnamomea	Activity	Inhibit adipogenesis, inflammation, and oxidative stress	NAFLD	[178]
	Expression	Inhibit oxidative stress and maintain mitochondrial homeostasis	Cardiac hypertrophy	[179]
Formononetin	Expression	Improve mitochondrial dysfunction and cardiac fibrosis	Cardiac fibrosis	[180]
SGLT2i	Activity and expression	Promote cardiac remodeling	Myocardial remodeling	[181]
Luteolin	Activity and expression	/	/	[182]
Inhibitor				
Disulfiram	Activity	Inhibit ALDH2-mediated endothelial-derived protective effect	RIPC	[183]
Daidzin	Activity	Prevent the development of aortic aneurysm or dissection	AAD	[184]
	Activity	Inhibit LDL-mediated smooth muscle cell apoptosis	Atherosclerosis	[74]
Cyanamide	Activity	Inhibit ALDH2-mediated cardioprotection	MI	[183]
Benomyl	Activity	Aggravate substantial loss in dopaminergic neurons	PD	[185]
Clinical trial				
ANS-6637	Activity	Phase 2	Alcohol use disorder	NCT04311294
Isoflavones	Activity	Phase 1	Alcohol metabolism	NCT02309801

*AAD* aortic aneurysm and dissection, *ALDH2* aldehyde dehydrogenase 2, *I/R* ischemia/reperfusion, *LUAD* lung adenocarcinoma, *MI* myocardial infarction, *NAFLD* non-alcoholic fatty liver disease, *PD* Parkinson’s disease, *RIPC* remote ischemic preconditioning, *LDL* low-density lipoprotein

### Transcriptional regulation of ALDH2

Notably, numerous studies have demonstrated that certain molecules can regulate ALDH2 expression and activity at the transcriptional level. The details of these molecules are summarized in Table 4 [75, 80, 98, 179, 186–194].

**Table 4:** Transcriptional regulators of ALDH2

Molecules	Effect and mechanism	Diseases	References
NFYA	Activate ALDH2 transcription by binding to the ALDH2 promoter region	Lung cancer	[186]
EHMT2	Inhibit ALDH2 transcription through H3K9me2 modification	Lung cancer	[186]
HNF4	Activate ALDH2 transcription by binding to the ALDH2 promoter region	Renal cell carcinomas	[187]
VHL	Activate ALDH2 transcription by upregulating HNF4 expression	Renal cell carcinomas	[187]
FOXM1	Activate ALDH2 transcription by binding to the ALDH2 promoter region	Hepatocellular carcinoma	[188]
CHKB-DT	Stabilize ALDH2 mRNA to maintain its transcription	Dilated cardiomyopathy	[189]
CREB1	Activate ALDH2 transcription by binding to the ALDH2 promoter region	Osteoarthritis; Lung adenocarcinoma	[80, 98]
SNW1-RXR	Activate ALDH2 transcription by binding to the ALDH2 promoter region	Cardiac hypertrophy	[179]
PPARβ/δ	Activate ALDH2 transcription (mechanism unclear)	Myocardial I/R injury	[190]
NRF2	Activate ALDH2 transcription (mechanism unclear)	Alcoholic liver disease	[191]
miR-34a	Inhibit ALDH2 transcription by binding to the 3’UTR of ALDH2 mRNA	Myocardial infarction	[192]
miR-27a-3p	Inhibit ALDH2 transcription by binding to the 3’UTR of ALDH2 mRNA	Obesity	[193]
miR-193b-3p	Inhibit ALDH2 transcription by binding to the 3’UTR of ALDH2 mRNA	Atherosclerosis	[75]
miR-224	Inhibit ALDH2 transcription by binding to the 3’UTR of ALDH2 mRNA	Lipid metabolism	[194]

*NFYA* nuclear transcription factor Y subunit alpha, *ALDH* aldehyde dehydrogenase 2, *EHMT2* euchromatic histone lysine methyltransferase 2, *HNF4* Hepatocyte nuclear factor 4,*VHL* von Hippel-Lindau protein, *FOXM1* forkhead box protein M1, *CHKB-DT* CHKB divergent transcript, *CREB1* cAMP-responsive element protein 1, *SNW1* SNW domain-containing protein 1, *RXR* retinoid X receptor, *PPARβ/δ* peroxisome proliferator-activated receptor beta/delta, *NRF2* nuclear factor erythroid 2-related factor 2, *I/R* ischemia/reperfusion

## Conclusions and perspectives

Dysfunctional autophagy and cell death have been confirmed as leading causes of the occurrence and development of multiple human diseases [1]. Understanding their underlying regulatory mechanisms and exploring their key regulators may shed light on new targets for therapeutic interventions. In this review, we outline the noncanonical function of ALDH2 in regulating autophagy and various types of cell death. Emerging evidence has confirmed that ALDH2 not only mediates the degradation of multiple aldehydes but also plays a unique role in modulating autophagy and cell death, thereby contributing to cellular homeostasis and tissue development [6, 10]. The complex cellular structure and function of ALDH2 are closely related to its location. Further exploration of differences in the spatial localization and function of ALDH2 within cells or organelles can provide new directions for elucidating its underlying mechanisms in regulating autophagy and cell death. Additionally, the connection between autophagy and different types of cell death appears to be important for cellular function and homeostasis. The occurrence and progression of many diseases are accompanied by dysfunctional autophagy and the activation of multiple cell death pathways. Therefore, targeting the regulation of autophagy or multiple cell death pathways through ALDH2 deserves further investigation, as it could yield promising approaches for treating human diseases.

Additionally, we highlight that ALDH2 deficiency and dysfunction are associated with a variety of pathological conditions. Notably, both ALDH2 deficiency and ALDH2 dysfunction can lead to a reduction in cellular ALDH2 activity, but these conditions should not be confused as a single pathology. For example, the dysfunction of ALDH2 caused by the rs671 mutation significantly inhibits autophagy and accelerates atherosclerotic progression by promoting the AMPK-mediated phosphorylation of ALDH2 and suppressing *ATG* gene transcription in a phosphorylated ALDH2-dependent manner. Interfering with ALDH2 expression has a protective effect on this process [42]. Therefore, exploring the exact role of ALDH2 in different pathological states is necessary to guide the development of ALDH2-based therapies for human diseases. Notably, the ALDH and carboxypeptidase families play important roles in detoxification, and their interaction has recently been reported in cancer [195, 196]. Specifically, carboxypeptidase A4 is strongly correlated with the cancer stem cell marker ALDH1A1 and could serve as a prognostic marker for ESCC [195]. These findings indicate that ALDH and carboxypeptidase proteins may act synergistically in disease pathogenesis, suggesting their combined targeting as a potential therapeutic approach worthy of further study.

Although significant advances have been made in understanding how ALDH2 regulates autophagy and cell death and its associated roles in human diseases, some issues remain to be elucidated. For example, why ALDH2 exhibits disparate functions within different organelles, and its core mechanisms need to be further explored. Is there a feedback regulatory mechanism, namely, autophagy-mediated regulation of ALDH2? Does ALDH2-dependent control of autophagy contribute to the maintenance of a stress threshold? How does ALDH2 sense cell death signals in response to different physiological or pathological conditions, and how does it determine cell fate? In addition to the above, does ALDH2 also regulate other cell death pathways? Are there any specific biomarkers for monitoring ALDH2-dependent autophagy and cell death? Can ALDH2 serve as an indicator of diagnosis or deterioration during disease, and should this be explored? How can the difference between enzymatic and nonenzymatic ALDH2 activity in human diseases be evaluated? How can ALDH2-dependent drugs for human diseases be developed?

## Data Availability

Not applicable.

## Abbreviations

4-HNE: 4-Hydroxy-2-nonenal
AAA: Abdominal aortic aneurysm
ACSL4: Acyl-CoA synthetase long-chain family member 4
AD: Alzheimer’s disease
ADCD: Autophagy-dependent cell death
AKI: Acute kidney injury
Akt: Threonine-serine protein kinase B
ALDH: Aldehyde dehydrogenase
AMPK: AMP-activated protein kinase
ATF6: Activating transcription factor 6
ATG: Autophagy-related
ATP6V0E2: ATPase H^+^ transporting V0 Subunit E2
BCL2: B-cell lymphoma 2
BECN1: Beclin1
CASPs: Caspases
cGAS: Cyclic guanosine monophosphate-adenosine monophosphate synthase
CHOP: C/EBP homologous protein
CREB1: CAMP-responsive element protein 1
DRP1: Dynamin-related protein 1
DSCR1-1: Down syndrome candidate region 1–1
eIF2α: Eukaryotic initiation factor 2 alpha
ER: Endoplasmic reticulum
ESCC: Esophageal squamous cell carcinoma
FUNDC1: FUN14 domain containing 1
GPX4: Glutathione peroxidase 4
GRP78: Glucose-regulating protein 78
GSDMD: Gasdermin-D
GSDME: Gasdermin-E
HDAC3: Histone deacetylase 3
HF: Heart failure
HMGCR: 3-Hydroxy-3-methylglutaryl-coenzyme A reductase
I/R: Ischemia/reperfusion
LPS: Lipopolysaccharide
IREα: Inositol-requiring enzyme 1α
JNK: C-Jun N-terminal kinase
LTC4: Leukotriene C4
MAPK: Mitogen-activated protein kinases
MGST2: Microsomal glutathione S-transferase 2
MI: Myocardial infarction
mTOR: Mechanistic target of rapamycin
NAD^+^: Nicotinamide adenine dinucleotide
NADPH: Nicotinamide adenine dinucleotide phosphate
NLRP3: Nucleotide-binding domain (NBD), leucine-rich repeat (LRR), and pyrin domain (PYD)-containing protein 3
PAD4: Peptidyl arginine deiminase 4
PERK: Pancreatic ER kinase (PKR)-like ER kinase
PGC-1α: Peroxisome proliferator-activated receptor gamma coactivator 1 alpha
PHB2: Prohibitin 2
PI3K: Phosphatidylinositol 3-kinase
PINK: PTEN-induced putative kinase 1
PKCε: Protein kinase C ε
PRKN: Parkin RBR E3 ubiquitin protein ligase
PTMs: Post-translational modifications
RCD: Regulated cell death
RIPK3: Receptor-interacting protein kinase 3
ROS: Reactive oxygen species
Ser: Serine
SIRT3: Sirtuin 3
STING: Stimulator of interferon genes
TBK1: TANK-binding kinase 1
Thr: Threonine
Trx-1: Thioredoxin-1
ULK: Unc-51-like kinase
UPR: Unfolded protein response
VPS: Vacuolar protein sorting
Wnt: Wingless and int-1
XBP-1: X-box binding protein-1
